# Bioorthogonal CRISPR/Cas9‐Drug Conjugate: A Combinatorial Nanomedicine Platform

**DOI:** 10.1002/advs.202302253

**Published:** 2023-07-23

**Authors:** Marcel Janis Beha, Joo‐Chan Kim, San Hae Im, Yunsu Kim, Seungju Yang, Juhee Lee, Yu Ri Nam, Haeshin Lee, Hee‐Sung Park, Hyun Jung Chung

**Affiliations:** ^1^ Department of Biological Sciences Korea Advanced Institute of Science and Technology Daejeon 34141 Republic of Korea; ^2^ Department of Chemistry Korea Advanced Institute of Science and Technology Daejeon 34141 Republic of Korea; ^3^ Graduate School of Nanoscience and Technology Korea Advanced Institute of Science and Technology Daejeon 34141 Republic of Korea

**Keywords:** bioorthogonal, cancer therapy, chemotherapeutic drugs, combinatorial delivery, CRISPR/Cas9, gene editing, nanomedicines, unnatural amino acids

## Abstract

Bioconjugation of proteins can substantially expand the opportunities in biopharmaceutical development, however, applications are limited for the gene editing machinery despite its tremendous therapeutic potential. Here, a self‐delivered nanomedicine platform based on bioorthogonal CRISPR/Cas9 conjugates, which can be armed with a chemotherapeutic drug for combinatorial therapy is introduced. It is demonstrated that multi‐functionalized Cas9 with a drug and polymer can form self‐condensed nanocomplexes, and induce significant gene editing upon delivery while avoiding the use of a conventional carrier formulation. It is shown that the nanomedicine platform can be applied for combinatorial therapy by incorporating the anti‐cancer drug olaparib and targeting the RAD52 gene, leading to significant anti‐tumor effects in BRCA‐mutant cancer. The current development provides a versatile nanomedicine platform for combination treatment of human diseases such as cancer.

## Graphical abstract

Here, a nanomedicine platform based on bioorthogonal CRISPR/Cas9‐drug conjugates, which can be self‐delivered in vivo and exert strong anti‐tumor effects upon gene editing and chemotherapy is developed. The current development provides a powerful strategy that can be widely applied for the therapy of human diseases such as cancer and genetic disorders.

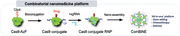

## 1 Introduction

Protein conjugation is an invaluable tool for the development of biopharmaceuticals, which can provide novel functions or enhance the efficacy of the therapeutic.^[^ ^1^, ^2^, ^3^ ^]^ Immunoconjugates, such as antibody‐drug conjugates (ADCs), and long‐acting protein formulations have been the major class of therapeutics that are generated by covalent conjugation of a protein with a secondary agent.^[^ ^4^, ^5^, ^6^, ^7^ ^]^ Conventionally, natural functional groups in cysteine or lysine residues of the protein have been generally used for modification, which shows clear limitations in versatility and quality control.^[^ ^5^, ^8^ ^]^ Bioorthogonal conjugation using chemoselective functionalities can expand the opportunities by introducing novel functions as well as multi‐modal activities to the platform.^[^ ^9^, ^10^, ^11^, ^12^ ^]^ Although shown promising for imaging and diagnostic applications,^[^ ^13^, ^14^ ^]^ bioorthogonal chemistry has been limitedly applied for biopharmaceutical development, due to the difficulty in achieving the desired additive effects while preserving the activity of the individual molecules.

The clustered regularly interspaced short palindromic repeat/CRISPR‐associated protein 9 (CRISPR/Cas9) system has emerged as an innovative tool for human gene therapy.^[^ ^15^, ^16^, ^17^ ^]^ However, in vivo CRISPR therapy has faced major hurdles due to poor delivery and low efficacy when applied to diseases involving progressive or complex microenvironments such as cancer.^[^ ^18^ ^]^ Although combinatorial therapy with conventional drugs can improve outcomes, treating the agents in separate formulations can lead to severe toxicity and diminish their synergistic effects.^[^ ^19^, ^20^, ^21^ ^]^ Delivery strategies for the CRISPR/Cas9 system based on lipids, polymers, and inorganic nanoparticles have been reported to induce significant gene editing efficiencies in vivo.^[^ ^22^, ^23^, ^24^, ^25^, ^26^ ^]^ However, the co‐delivery of the gene editing machinery and chemical drugs in a single formulation has been challenging due to the distinct physicochemical properties of the cargo.^[^ ^18^ ^]^

In vivo gene editing of cancer cells has been intensively investigated in the past several years. Previous studies on the delivery of the CRISPR system to tumors have involved the disruption of oncogenes (e.g., plk‐1, KRAS),^[^ ^27^, ^28^ ^]^ or immunotherapy targets.^[^ ^29^, ^30^ ^]^ For cancer, treatment of a chemotherapeutic drug combined with gene therapy can give rise to synergistic effects and has been shown promising for oligonucleotide therapy (e.g., siRNA, ASO), with clinical trials ongoing. However, silencing oligonucleotides show limitations for treating cancer due to the transient effects, requiring repeated administrations and leading to a high chance of recurrence.^18^ On the other hand, permanent genome editing of cancer cells can provide great benefits in treatment outcomes by the prolonged effects. However, the challenges in efficient penetration and co‐localization of the gene editing machinery in tumor tissues must be overcome.

Covalent conjugation has been suggested as an alternative approach for the delivery of the CRISPR machinery.^[^ ^30^, ^31^, ^32^ ^]^ Previous studies have reported the conjugation of Cas9 with carrier materials such as polymers and peptides via the natural cysteine groups of the protein, resulting in effective delivery and gene editing in bacteria and cells.^[^ ^30^, ^33^, ^34^, ^35^ ^]^ However, advanced conjugation methods are required to improve the versatility and diversity of Cas9 functionalization. Recently, the site‐specific incorporation of Cas9 with an azide‐containing unnatural amino acid has been introduced, for subsequent chemical modification via strain‐promoted [3+2] azide‐alkyne cycloaddition (SPAAC) coupling.^[^ ^32^, ^36^, ^37^ ^]^ Chemically modified Cas9 with single‐guide RNA (sgRNA) or donor DNA oligonucleotides was delivered via electroporation and was able to induce enhanced gene editing efficiencies *ex vivo*. However, in vivo delivery of site‐specifically modified Cas9, and furthermore, their co‐delivery with secondary molecules, such as chemotherapeutic drugs, has yet to be explored.

**Figure 1:**
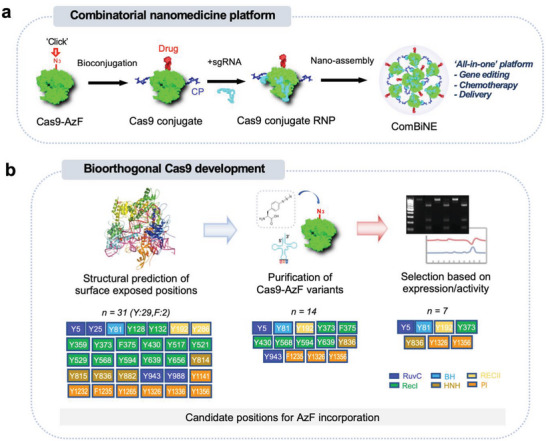
Schematic for the combinatorial nanomedicine platform based on bioorthogonal CRISPR/Cas9 conjugates. a) Development of ComBiNE based on the conjugation of Cas9‐AzF with drug molecules and carrier polymer (CP), and subsequent complexation with sgRNA, forming nano‐assembled RNP complexes. b) Production and selection process of Cas9‐AzF variants. Selected candidate Tyr and Phe positions for AzF incorporation are shown in colored boxes according to the Cas9 domain.

Here, we developed a nanomedicine platform based on a bioorthogonal CRISPR/Cas9 conjugate that can self‐deliver the gene editing machinery and a chemotherapeutic drug in a single formulation. We generated a series of bioorthogonal Cas9 variants (Cas9‐AzF) by site‐specific incorporation of 4‐azido‐l‐phenylalanine (AzF), which were subsequently conjugated with a chemotherapeutic agent and a carrier polymer (CP), resulting in their self‐condensation into nano‐sized complexes (**Figure** **1**a). The formulation, termed combinatorial and bioorthogonal nano‐editing complex (ComBiNE), was applied as a therapeutic by incorporation of the drug olaparib and targeting the RAD52 gene, and could substantially suppress tumor growth in vivo in a BRCA‐mutant breast cancer model. To the best of our knowledge, this is the first study to report a covalent CRISPR/Cas9‐drug conjugate platform that can be self‐delivered and lead to therapeutic efficacy in vivo. The current development provides a powerful strategy for treating various human diseases such as cancer and genetic disorders.

## 2 Results and Discussion

### 2.1 Bioorthogonal CRISPR/Cas9 Development

**Figure 2:**
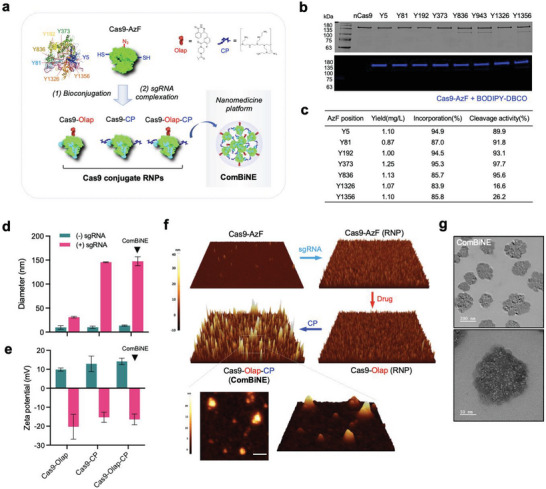
Generation of ComBiNE based on self‐condensation of CRISPR/Cas9 conjugates. a) Schematic for bioconjugation of Cas9‐AzF with either olaparib (Cas9‐Olap), CP (Cas9‐CP), or both olaparib and CP (Cas9‐Olap‐CP), and subsequent complexation with sgRNA, resulting in Cas9 conjugate RNPs. Particularly, Cas9‐Olap‐CP RNPs spontaneously undergo nano‐assembly to form ComBiNE. b) Characterization of Cas9‐AzF variants for different AzF positions by SDS‐PAGE (top), and by reaction with DBCO‐BODIPY to confirm AzF incorporation (bottom). c) Summary showing purification yields, AzF incorporation efficiencies, and cleavage activities of the Cas9‐AzF variants. d–g) Physicochemical characterization of ComBiNE and Cas9 conjugates prepared with the Y373 variant. d) DLS and e) zeta potential measurements of Cas9 conjugates in presence and absence of sgRNA (molar ratio of Cas9:sgRNA = 1:1). f) AFM and g) TEM analyses of ComBiNE and control formulations.

We generated a bioorthogonal form of the CRISPR/Cas9 system, wherein the Cas9 protein was engineered by site‐specific incorporation of AzF to various amino acid positions. We sought to select the optimal Cas9‐AzF variant based on purification yields and maintenance of endonuclease function of the Cas9 machinery. We considered only Tyr and Phe residues which are structurally similar to AzF, as mutable sites. Structural prediction led to a total of thirty‐one Tyr and Phe residues in the six different Cas9 domains: RuvC, Bridge Helix (BH), RECI, RECII, HNH, and PAM‐interacting (PI) domain (Figure 1b).^[^ ^38^, ^39^ ^]^ Among the 31 positions, we succeeded in the purification of Cas9‐AzF variants for 14 positions. We then selected a total of seven variants with AzF incorporated into different Cas9 domains that exhibited stable protein expression and high purity: Y5 (RuvC), Y81 (BH), Y192 (RECII), Y373 (RECI), Y836 (HNH), Y1326 (PI) and Y1356 (PI). We produced the seven Cas9‐AzF variants in large quantities for further characterization and bioconjugation (**Figure** **2**a). The reaction of Cas9‐AzF with dibenzocyclooctyne (DBCO)‐modified fluorophore resulted in fluorescence coupling of the protein via SPAAC, confirming the incorporation of the azido group (Figure 2b). The purification yields of the Cas9‐AzF variants for positions Y5, Y81, Y192, Y373, Y836, Y1326, and Y1356 appeared to be between 0.87 to 1.25 mg L^−1^, with the Y373 variant showing the highest yield (Figure 2c). Quantification of the azido groups by a fluorescence tagging method demonstrated conjugation efficiencies of between 83.9% and 95.3%, further confirming the successful incorporation of AzF into the protein. Examining the endonuclease activity of the different Cas9‐AzF variants on target DNA cleavage showed efficiencies of 89.9% to 97.7% for positions Y5, Y81, Y192, Y373, and Y836, with the Y373 variant showing the highest efficiency (Figure 2c and Figure S1, Supporting Information). Clearly, the incorporation of AzF into Cas9 for these positions did not result in a major loss of endonuclease function, as can be inferred by comparing the values with native Cas9 (98.4%). However, cleavage efficiencies for the Y1326 and Y1356 variants appeared to be 16.6% and 26.2%, respectively, suggesting that incorporating AzF within the PI domain led to a substantial loss of endonuclease activity, possibly by reducing the binding affinity of Cas9 with the target DNA motif.^[^ ^39^ ^]^ In summary, these results demonstrate the importance of selecting the appropriate position for incorporating AzF into Cas9, as mutations at some sites can severely disrupt enzyme function. Since the Cas9‐AzF variant for position, Y373 showed the highest purification yield and maintenance of endonuclease activity, we selected the Y373 variant for further experiments.

### 2.2 Development of ComBiNE, a Self‐Condensed Nanomedicine Platform

We next investigated if the bioorthogonal CRISPR/Cas9 (Cas9‐AzF) can be engineered into a self‐delivered formulation, ComBiNE. We hypothesized that multi‐functionalization of Cas9‐AzF with a drug and CP, and its subsequent complexation with sgRNA would result in self‐condensation of the RNPs into nano‐sized complexes. We generated ComBiNE based on multi‐functionalization of the Cas9 protein by conjugation of 1) one type of molecule (e.g., drug) via bioorthogonal reaction with the azido groups, and 2) another type of molecule (e.g., polymer) via sulfhydryl groups of natural cysteines, or vice versa. Using azide‐DBCO and cysteine‐maleimide chemistry, we created Cas9 modified with the anti‐cancer drug olaparib and low molecular weight branched polyethylenimine, respectively, to produce Cas9‐Olap‐CP (Figure 2a and Figure S2, Supporting Information). Olaparib is an FDA‐approved anti‐cancer drug that inhibits the nuclear poly(ADP‐ribose) polymerase (PARP) and has been reported to be effective against BRCA1/BRCA2 mutant cancers of the ovaries, breast, and prostate.^[^ ^40^, ^41^ ^]^ Polyethylenimine is a widely used delivery carrier that shows efficient transfection, but in the meantime shows serious cytotoxicity when complexed with nucleic acids in free form, limiting their applications for in vivo delivery. Although showing limitations when used in free form, it has been reported that polyethylenimine can be versatilely engineered into nano‐formulations and can serve as excellent drug carriers.^[^ ^42^, ^43^ ^]^ We anticipated that conjugation of an extremely small amount of CP (< 2.5 wt.% of cargo) onto Cas9 molecules could enable self‐condensation of the RNPs into nano‐sized complexes.^[^ ^30^ ^]^ Quantification of residual azido‐ and sulfhydryl groups confirmed the successful conjugation of both olaparib and CP onto Cas9 (Figure S3, Supporting Information). Matrix‐assisted laser desorption ionization‐time of flight (MALDI‐TOF) spectrometry analysis showed the appearance of a broad peak width, as well as a significant peak shift for the Cas9‐Olap conjugates (m/z 163 379.00) compared to unmodified Cas9‐AzF (m/z 162 592.18), confirming the successful conjugation of the drug molecule to the Cas9 protein (Figure S4, Supporting Information). We then examined if the Cas9‐Olap‐CP conjugates can undergo self‐condensation and nano‐assembly, to form ComBiNE. We observed that adding sgRNA to Cas9‐Olap‐CP and Cas9‐CP induced the formation of nano‐sized particle complexes with hydrodynamic diameters of 147.5 nm and 145.5 nm, respectively, whereas Cas9‐Olap lacking CP formed smaller complexes of 31.1 nm (Figure 2d). Further characterization of the self‐condensation process was performed by physicochemical analyses of ComBiNE in various conditions. The hydrodynamic sizes of ComBiNE (Cas9:sgRNA = 1:1) gradually increased upon incubation time, showing values of 168.9 nm at 1 min, 317.9 nm at 30 min, and up to 469.7 nm at 60 min (Figure S5, Supporting Information). Polydispersity indexes (PDI) showed values of 0.28 at Cas9:sgRNA molar ratios of 1:0.5–1:1, and the values slightly increased to 0.33–0.38 for ratios of 1:3–1:5. Meanwhile, Cas9‐Olap RNP complexes as the control showed sizes of < 21.0 nm, throughout the incubation period. Taking into account that similar values were observed for Cas9‐Olap‐CP and Cas9‐CP in the presence of sgRNA, the results demonstrate that the conjugation of CP onto Cas9 was the key factor in inducing self‐condensation of the RNPs into nano‐complexes with the optimal size for delivery.^[^ ^44^ ^]^ In the absence of sgRNA, hydrodynamic diameters appeared to be only 13.5 nm for Cas9‐Olap‐CP, proving that the interaction of sgRNA with the cationic polymer on the protein was the driving force in nano‐assembly of the RNPs. Increases in zeta potential values of RNPs for Cas9‐Olap‐CP (−16.4 mV) compared to Cas9‐Olap (−20.3 mV) demonstrate that differences in surface charge can affect the tendency in nano‐complex formation (Figure 2e). Analysis by AFM revealed the presence of nano‐sized, particle‐like structures for ComBiNE, which were obviously distinct from the Cas9‐Olap RNPs lacking CP conjugation (Figure 2f and Figure S6, Supporting Information). TEM analysis of ComBiNE showed the presence of nano‐sized, grape‐like structures, with average sizes of 146.6 nm, wherein large numbers of smaller particles (<10 nm) resembling single Cas9 molecules were observed (Figure 2g). Accordingly, we anticipated that the ComBiNE formulation can be an effective nanomedicine platform for combinatorial delivery.

### 2.3 Examination of ComBiNE for Self‐Delivery and Gene Editing In Vitro

**Figure 3:**
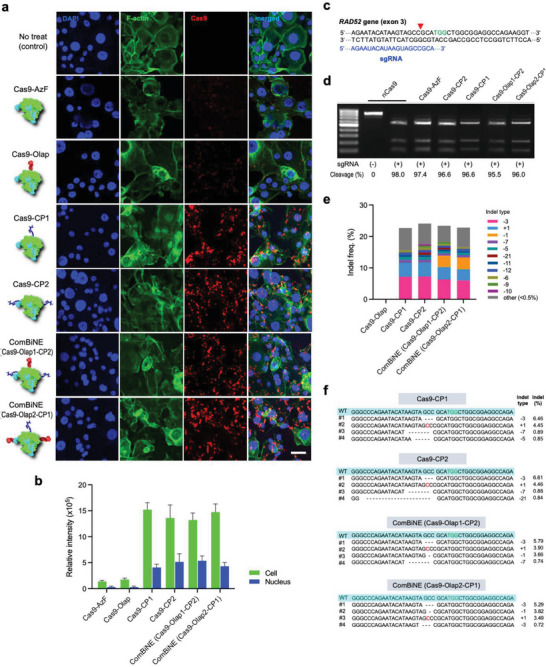
Delivery of ComBiNE for gene editing in vitro. ComBiNE and control formulations were formed using conjugates of the Y373 variant, and complexation with sgRNA targeting RAD52 (molar ratio of Cas9:sgRNA = 1:1). a) Confocal microscopy of BRCA1‐mutant HCC1937 breast cancer cells treated with ComBiNE or control formulations for 4 h at 500 nM Cas9 (blue: DAPI, green: F‐actin, red: Cas9 labeled with AF647; scale bar: 50 µm). b) Quantification of uptake efficiencies by image analysis of (a). c) sgRNA design for the RAD52 gene (blue: sgRNA sequence, black: target sequence of RAD52 gene, green: PAM sequence, red arrowhead: cleavage site). d) Cleavage activities examined by treating Cas9 conjugate RNPs to RAD52 target DNA, and analysis of cleaved DNA fragments. e) Gene editing efficiencies, and f) representative sequence data (green: PAM, ‐: deletion, red: insertion, WT: wild‐type) of HCC1937 cells treated with ComBiNE and control formulations for 48 h at 500 nM Cas9, and targeted deep sequencing analysis. For (e), indel types with frequency values of > 0.5% for at least one formulation are shown in different colors in the stacked bars.

We evaluated the potential of ComBiNE for delivery to cells without the use of an external carrier, and to induce gene editing in vitro. We prepared ComBiNE based on two different forms of Cas9‐Olap‐CP (Cas9‐Olap1‐CP2 and Cas9‐Olap2‐CP1), depending on the functional groups used for conjugation of olaparib and CP. RNPs were also prepared using two different forms of Cas9‐CP (Cas9‐CP1 and Cas9‐CP2), as controls. When treated to BRCA1‐mutant HCC1937 breast cancer cells, both ComBiNE and Cas9‐CP RNPs were efficiently internalized, demonstrating that ComBiNE could serve as a self‐delivered platform (**Figure** **3**a, b). The levels of uptake for ComBiNE and Cas9‐CP RNPs did not significantly vary depending on the functional groups used for conjugation. ComBiNE was also substantially internalized into the nucleus, meeting the requirements of sub‐cellular localization for function of the gene editing machinery. On the other hand, Cas9‐Olap and unmodified Cas9‐AzF RNPs did not show significant uptake. These results demonstrate that conjugation of only 1–2 molecules of CP on Cas9 led to the successful uptake of the RNPs into cells, without the use of an additional carrier.

We next assessed if ComBiNE formed with sgRNA targeting the RAD52 gene (Figure 3c) can induce genetic changes upon self‐delivery to target cells in vitro. Examining the endonuclease activity revealed that no significant loss in function occurred for the Cas9‐Olap‐CP conjugates (>95.5% cleavage) (Figure 3d). Targeted deep sequencing analyses of HCC1937 cells after treatment revealed that ComBiNE significantly induced indels at the target locus of the RAD52 gene (Figure 3e). The type of functional group used (azido or sulfhydryl group) for conjugation of olaparib and CP to Cas9 did not significantly affect the gene editing efficiencies; that is, similar indel frequencies were shown for ComBiNE based on Cas9‐Olap1‐CP2 and Cas9‐Olap2‐CP1. The results for Cas9‐CP RNPs were also similar to ComBiNE, demonstrating that gene editing occurred regardless of the presence or absence of olaparib. On the other hand, RNPs of unmodified Cas9‐AzF and Cas9‐Olap were not able to induce gene editing (< 0.01%), proving that the conjugation of CP was essential for the delivery of Cas9. Representative sequences from HCC1937 cells treated with ComBiNE included three‐base deletions with the highest frequencies (> 5.0%) as well as single‐base deletions and single‐base insertions (> 3.0%) (Figure 3f and Figure S7, Supporting Information). Similar patterns in sequences were shown for Cas9‐CP RNPs and ComBiNE. Treatment of Cas9‐CP RNPs prepared with non‐target sgRNA to HCC1937 cells resulted in cell viability of > 99%, demonstrating the low toxicity of CP upon conjugation onto the Cas9 protein (Figure S8). In contrast, complexes of free polyethylenimine and Cas9‐AzF RNPs showed high cytotoxicity (≈7% cell viability for 24 h treatment). Taken together, these results demonstrate the potential of ComBiNE as an effective and self‐delivered gene editing platform.

### 2.4 Functional Validation of CRISPR/Cas9‐Drug Conjugate for Combination Treatment in Cancer Cells

**Figure 4:**
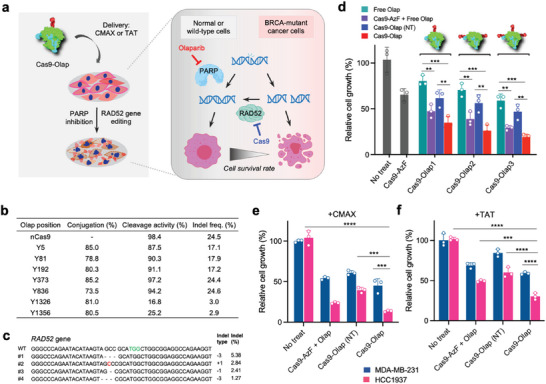
Functional validation of CRISPR/Cas9‐drug conjugates for combination treatment in cancer cells in vitro. a) Schematic showing the principle of synthetic lethality induced by Cas9‐Olap conjugate RNPs, based on the function of olaparib and RAD52 gene editing in BRCA‐mutant cancer. b) Characterization of Cas9‐Olap conjugates prepared with the various Cas9‐AzF variants: olaparib conjugation efficiencies, cleavage activities, and indel frequencies. c) Representative sequence data by targeting deep sequencing of HCC1937 cells treated with Cas9‐Olap RNPs complexed with CMAX for 48 h at 500 nM Cas9. d–f) Combinatorial effect of treating Cas9‐Olap RNPs prepared with the Y373 variant, on anti‐proliferation of cells using conventional carriers for 72 h (500 nM Cas9; *n* = 3, One‐way ANOVA, ^**^ *p* < 0.01, ^***^ *p* < 0.001, ^****^ *p* < 0.0001). d) Effect of Cas9‐Olap RNPs prepared by conjugation of olaparib via azido (Cas9‐Olap1), sulfhydryl (Cas9‐Olap2), and both azido and sulfhydryl (Cas9‐Olap3) groups, complexed with CMAX and treated to HCC1937 cells. e,f) Comparison of the anti‐proliferation effect in HCC1937 and MDA‐MB‐231 cells using e) CMAX, and f) TAT peptide (molar ratio of Cas9:TAT = 1:10) as the carrier. Concentration of free olaparib was adjusted to the conjugated olaparib concentration for the corresponding Cas9‐Olap conjugate in each group. Cas9‐Olap conjugates were complexed with RAD52 sgRNA at a molar ratio of 1:1.

We examined if the co‐delivery of Cas9 and olaparib as covalent conjugates can induce a combinatorial effect on anti‐proliferation of cancer cells in vitro. We expected the Cas9‐Olap conjugates complexed with sgRNA targeting the RAD52 gene to synergistically induce cancer cell death by simultaneously targeting the DNA repair pathway, and that this effect can be enhanced in BRCA‐mutant cells due to their high sensitivity to olaparib (**Figure** **4**a).^[^ ^45^, ^46^ ^]^ Tumor cells are said to exhibit high levels of DNA damage, and RAD52‐dependent homologous recombination (HR) plays an important role in DNA repair for cell survival. We prepared the Cas9‐Olap conjugates by reacting either the azido groups of AzF (Cas9‐Olap1), sulfhydryl groups of cysteine (Cas9‐Olap2), or both the azido and sulfhydryl groups (Cas9‐Olap3) of Cas9 with olaparib. Characterization of the Cas9‐Olap conjugates revealed 73.5% to 85.2% conjugation efficiencies (Figure 4b). For variants Y5, Y81, Y192, Y373, and Y836, endonuclease activities were well maintained and were able to induce significant gene editing of RAD52 upon delivery using CRISPRMAX (CMAX), a conventional lipofectamine formulation (Figure 4b,c and Figures S9–11, Supporting Information). Importantly, the Cas9‐Olap conjugates targeting RAD52 were effective in inhibiting the growth of BRCA1‐mutant HCC1937 breast cancer cells when delivered with CMAX, whereas the Cas9‐Olap RNPs with control sgRNA (NT: non‐target) showed modest effects in anti‐proliferation (Figure 4d). All forms of the conjugates (Cas9‐Olap1, Cas9‐Olap2, and Cas9‐Olap3) induced significant cell death, while Cas9‐Olap3 was shown to be the most effective (19.3% relative growth), due to the presence of up to three olaparib molecules per Cas9 molecule. The Cas9‐Olap conjugates showed slight enhancements compare to treating a mixture of Cas9‐AzF RNPs and free olaparib, demonstrating the advantage of the conjugate platform. We note that free olaparib concentrations were adjusted to the levels of the conjugated olaparib on the corresponding Cas9‐Olap for each treatment group.

We assessed if the strong anti‐proliferation effect of Cas9‐Olap occurred by the presence of the BRCA mutation. For BRCA wild‐type cells (MDA‐MB‐231), Cas9‐Olap delivered with CMAX reduced cell growth, but to a milder extent that was not as strong as for HCC1937 cells (Figure 4e). The results imply that the Cas9‐Olap conjugates targeting RAD52 synergistically induced anti‐proliferation of cells, which was further enhanced in BRCA‐mutant cells by a dual synthetic lethality effect.^[^ ^45^ ^]^ Delivery of the Cas9‐Olap RNPs using another conventional carrier, the TAT peptide (GRKKRRQRRRPQ),^[^ ^47^ ^]^ also confirmed that the conjugates could act as an effective combinatorial agent (Figure 4f). Characterization of the Cas9‐Olap conjugate RNPs complexed with the TAT peptide revealed physicochemical properties that met the criteria for effective delivery (Figures S12–15, Supporting Information) and showed efficient internalization and gene disruption in cells (Figures S16 and S17, Supporting Information). The results demonstrate the potency of the Cas9‐Olap conjugates and their general applicability with various conventional carriers for delivery. Furthermore, the strategy of inducing synthetic lethality can reduce side effects that may occur due to the off‐target delivery of the CRISPR/Cas9 system and olaparib to normal cells.^[^ ^48^ ^]^

### 2.5 Evaluation of ComBiNE upon Self‐Delivery and Anti‐Proliferation of Cancer Cells In Vitro

**Figure 5:**
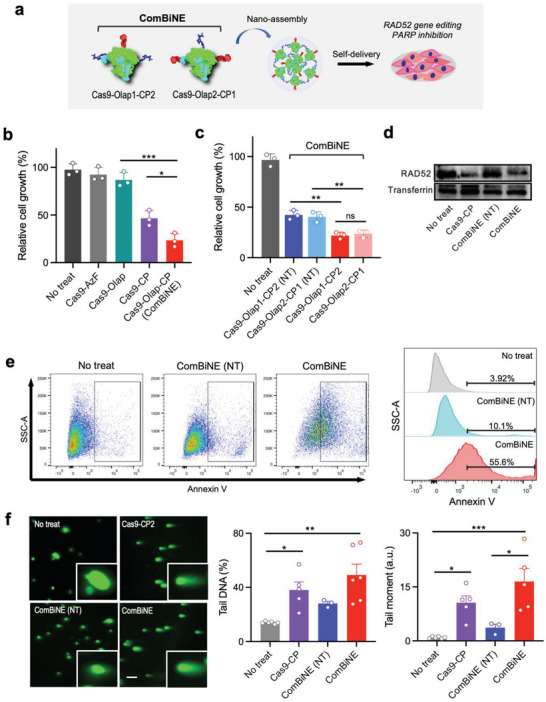
Evaluation of ComBiNE as a self‐delivered nanomedicine platform in cancer cells in vitro. a) Schematic showing the different forms of the ComBiNE platform and delivery to cells. ComBiNE can be prepared from conjugation of Cas9‐AzF with olaparib and CP via the azido and sulfhydryl groups, respectively (Cas9‐Olap1‐CP2), or vice versa (Cas9‐Olap2‐CP1). b,c) Anti‐proliferation effect upon treatment of ComBiNE and control formulations including RAD52 or control sgRNA (NT: non‐target) to HCC1937 cells for 72 h (*n* = 3, One‐way ANOVA, ^*^ *p* < 0.05, ^**^ *p* < 0.01, ^***^ *p* < 0.001). b) Demonstration of the synergistic effect of ComBiNE compared to Cas9‐CP and Cas9‐Olap conjugate RNPs. c) Comparison of ComBiNE prepared from Cas9‐Olap1‐CP2 and Cas9‐Olap2‐CP1 conjugates. d–f) Functional evaluation of ComBiNE (Cas9‐Olap1‐CP2). d) Western blot analysis showing the expression of RAD52, e) apoptosis levels measured by Annexin V assay, and f) DNA damage measured by the alkaline comet assay, in HCC1937 cells treated with ComBiNE for 48 h (One‐way ANOVA, ^*^ *p* < 0.05, ^**^ *p* < 0.01, ^***^ *p* < 0.001). ComBiNE formulations were prepared with the Y373 variant, and RAD52 sgRNA (molar ratio of Cas9:sgRNA = 1:1), followed by treatment at 500 nM Cas9.

We next evaluated the functional efficacy of ComBiNE as a self‐delivered formulation for suppressing the growth of cancer cells in vitro. To generate ComBiNE, we prepared conjugates of the Cas9 protein by reaction of 1) olaparib via bioorthogonal reaction with the azido groups, and 2) CP via sulfhydryl groups of natural cysteines, or vice versa. Although conjugation can also be performed on natural amine and carboxyl groups, in addition to the sulfhydryl groups, the presence of 150 amine groups and 207 carboxyl groups in the Cas9 protein will result in random conjugation at different sites. Here, the Cas9‐Olap‐CP conjugates were prepared by the attachment of olaparib and CP on the azido and sulfhydryl groups of Cas9, respectively, producing Cas9‐Olap1‐CP2, or vice versa to produce Cas9‐Olap2‐CP1. The conjugates were then complexed with RAD52 sgRNA to form ComBiNE (**Figure** **5**a). Delivery of ComBiNE significantly reduced the growth of HCC1937 cells, to an extent that was distinct from the results for Cas9‐CP RNPs showing a mild reduction (Figure 5b). The effect of treating a mixture of Cas9‐CP RNPs and free olaparib was significantly stronger than treating free olaparib alone, confirming the additive effect of RAD52 gene editing (Figure S18, Supporting Information). ComBiNE prepared with the different forms of conjugates (Cas9‐Olap1‐CP2 and Cas9‐Olap2‐CP1) appeared to have similar effects on cell growth (Figure 5c). Treating ComBiNE formed with control sgRNA (NT) led to mild anti‐proliferation effects, that can be attributed to the effect of solely the conjugated olaparib. The strong anti‐proliferation achieved by ComBiNE can be described as the synergistic effect of RAD52 gene disruption and inhibition of PARP1. Western blot analysis demonstrated that gene editing mediated by ComBiNE and Cas9‐CP conjugate RNPs led to a substantial reduction in RAD52 expression in HCC1937 cells (Figure 5d). Delivery of ComBiNE to MDA‐MB‐231 cells also resulted in a significant reduction in RAD52 levels (Figure S19, Supporting Information). On the other hand, ComBiNE formed with control sgRNA (NT) did not suppress RAD52 expression in either cell line. ComBiNE also significantly increased levels of late apoptosis in HCC1937 cells, compared to ComBiNE (NT) as the control (Figure 5e). Results from the alkaline comet assay revealed that the delivery of ComBiNE to HCC1937 cells substantially increased the number of comet‐positive cells, shown by the percentage of tail DNA (%) and tail moment, indicating high levels of double‐strand DNA breaks (Figure 5f). Cas9‐CP RNPs also induced significant levels of DNA damage, which were not as high as ComBiNE, due to the absence of olaparib. Treating ComBiNE (NT) resulted in lower values, revealing that RAD52 gene editing played a major role in inducing DNA damage. In MDA‐MB‐231 cells, lower values in tail DNA (%) and tail moment were observed for ComBiNE treatment compared to HCC1937 cells, which can be due to the superior DNA repair activity in BRCA wild‐type cells (Figure S17, Supporting Information). In sum, we revealed that the ComBiNE platform could effectively inhibit the growth of tumor cells, mediated by RAD52 gene editing and PARP inhibition, leading to cellular DNA damage and apoptosis.

### 2.6 In Vivo Efficacy of ComBiNE for Combinatorial Therapy in a BRCA‐Mutant Breast Cancer Model

**Figure 6:**
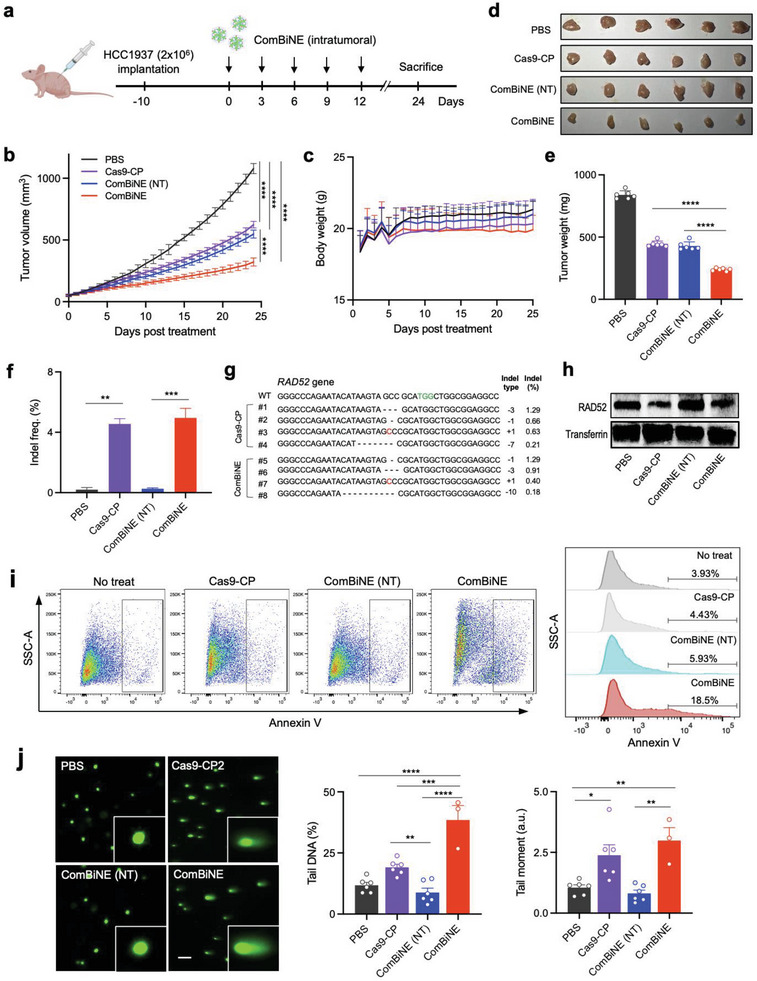
In vivo delivery and efficacy of ComBiNE in a BRCA‐mutant breast cancer model. a) Schematic of the timeline for tumor implantation and treatment of ComBiNE. b) Tumor growth curve based on daily measurements of tumor volumes (*n* = 6–8, ^****^ *p* < 0.0001), and c) average body weights of mice, from day 0 to day 24. d) Tumor images and (e) tumor weights (*n* = 6, ^****^ *p* < 0.0001) measured after sacrificing mice on day 24. f) Gene editing efficiencies (*n* = 2, ^**^ *p* < 0.01, ^***^ *p* < 0.001) and g) representative sequence data (green: PAM, ‐: deletion, red: insertion) by analyses of tumor tissues after sacrificing mice on day 5. Examination of h) RAD52 expression by western blot analysis, i) apoptosis levels by the Annexin V assay, and j) cellular DNA damage by the comet assay of excised tumor tissues on day 24 (^*^ *p* < 0.05, ^**^ *p* < 0.01, ^***^ *p* < 0.001, ^****^ *p* < 0.0001). ComBiNE (Cas9‐Olap1‐CP2) formulations were prepared with conjugates of the Y373 variant and either RAD52 sgRNA or control (NT) sgRNA (molar ratio of Cas9:sgRNA = 1:1), and locally administered at 25 µg of Cas9 per injection. One‐way ANOVA was performed to obtain *P* values and determine statistical significance.

We tested the in vivo efficacy of ComBiNE by combinatorial delivery and RAD52 gene editing in a BRCA‐mutant breast cancer model in mice. ComBiNE including RAD52 sgRNA was locally administered into HCC1937 tumors by repeated injections and tumor growth was monitored (**Figure** **6**a). We observed that treating ComBiNE targeting RAD52 resulted in major suppression of tumor growth, with a 70.3% reduction in tumor volume (322 mm^3^) compared to that of the PBS control (1079 mm^3^) on day 24 after the first treatment (Figure 6b). On the other hand, ComBiNE including control sgRNA (NT) led to only 49.2% reduction in tumor volume (548 mm^3^) compared to the PBS control. Treatment of Cas9‐CP RNPs also resulted in a mild reduction of tumors (42.1% vs PBS control, 625 mm^3^). Measuring the average body weights of mice showed minimal changes (< 6%) throughout the monitored period, indicating that no significant toxicity or side effects occurred in response to treatment (Figure 6c). Average tumor weights measured on day 24 appeared to be 240 mg for the ComBiNE treatment group, whereas values for Cas9‐CP, ComBiNE (NT), and PBS control were 445, 428, and 839 mg, respectively (Figure 6d,e). The results demonstrate the strong anti‐tumor effect of ComBiNE, due to the combination of RAD52 gene editing and function of olaparib. The observation that tumor growth was suppressed until 12 days after the last injection revealed that the effects of combinatorial treatment were sustained in vivo.

We further characterized the effect of ComBiNE in tumors by examining gene editing efficiencies. Targeted deep sequencing analyses of tumor tissues harvested on day 5 showed that both ComBiNE and Cas9‐CP RNPs led to significant gene editing of RAD52, as the indel frequency appeared to be 4.95% and 4.55%, respectively (Figure 6f). These results demonstrate the strong delivery potential of ComBiNE and Cas9‐CP RNPs, despite the challenge in penetration of the large and hydrophilic RNP complexes into tumors. On the other hand, ComBiNE (NT) did not induce significant gene editing, confirming the selectivity of RAD52 editing by ComBiNE in vivo. Analyses of tumors after sacrificing the mice at day 24 resulted in indels of <3% for both ComBiNE and Cas9‐CP treatment, indicating that a substantial portion of gene‐edited cells may have been removed during tumor growth (Figure S21, Supporting Information). Representative sequences from tumors for the ComBiNE and Cas9‐CP treatment group showed similar sequencing profiles, which included single‐base (> 0.6%) and three‐base (> 0.9%) deletions at the target locus of the RAD52 gene (Figure 6g). Western blot analysis proved that gene editing by ComBiNE and Cas9‐CP led to significantly reduced expressions of RAD52, while ComBiNE (NT) did not result in a significant decrease (Figure 6h).

We next validated the combinatorial effect of ComBiNE by functional analyses of the tumor tissues. Levels of apoptosis and cellular DNA damage were examined on tumor tissues harvested on day 24. ComBiNE treatment resulted in relatively high levels of late apoptosis in tumors, while ComBiNE (NT) and Cas9‐CP did not lead to significant apoptosis levels (Figure 6i). The high rate of apoptosis for the ComBiNE group confirms the combinatorial effect of RAD52 gene editing and olaparib in vivo.^[^ ^45^ ^]^ The alkaline comet assay revealed that ComBiNE treatment strongly induced DNA damage, whereas Cas9‐CP resulted in modest effects (Figure 6j). ComBiNE (NT) showed lower levels of DNA damage that were similar to the PBS control. The higher rates of apoptosis and cellular DNA damage for the ComBiNE treatment group can be correlated with the tumor growth reductions and gene editing results described above. In sum, we demonstrated the in vivo efficacy of the ComBiNE system as a therapeutic platform, which can efficiently disrupt the RAD52 gene and inhibit PARP1, resulting in significant anti‐tumor effects. ComBiNE is formed via the conjugation of an extremely small amount of carrier (<2.5 wt.% of Cas9), an amount that is 400 times reduced compared to the conventional nano‐complex formulations using free polyethylenimine.^[^ ^34^, ^49^ ^]^

Although some previous studies have demonstrated systemic delivery of the CRISPR/Cas9 system to tumors based on conventional nano‐formulations, the efficiency of delivering the RNPs, in general, has been extremely low.^[^ ^50^, ^51^ ^]^ In the clinic, intratumoral delivery has been a widely used route for adenovirus‐based therapy.^[^ ^52^ ^]^ Here, we show the efficacy of the ComBiNE platform through an intratumoral route, which can bring a substantial impact on cancer treatment in the clinic. To expand the opportunity of applying the ComBiNE platform, a formulation capable of systemic delivery (e.g., via intravenous administration) will be desired, which will confront challenges in prolonging in vivo stability and enhancing accumulation at the tumor tissue. Nonetheless, we anticipate that the ComBiNE platform can provide a major breakthrough in the treatment of various types of cancers and genetic diseases.

## 3 Conclusion

This study introduces the development of ComBiNE, a combinatorial and self‐delivered platform of the CRISPR/Cas9 machinery and chemotherapeutic drug. We demonstrate that ComBiNE is efficacious in a BRCA‐mutant breast cancer model, by editing the RAD52 gene and inhibiting PARP, leading to substantial anti‐tumor effects in vivo. The amount of synthetic carrier material used in the formulation is ≈400‐fold less than the amount in conventional nano‐complex formulations and therefore can minimize carrier toxicity and side effects in vivo. Our strategy can easily be extended to various chemotherapeutic agents and gene editing pairs. Furthermore, the bioorthogonal and site‐specific conjugation platform used for the CRISPR/Cas9‐drug conjugates can provide a versatile tool for biopharmaceutical development, such as ADCs, which require high‐quality control and versatility. Other advantages of the conjugate system are the controlled diffusion and distribution patterns of the chemotherapeutic drug, possibly reducing cytotoxicity. In sum, ComBiNE, as an all‐in‐one, multi‐functional platform enabling delivery, gene editing, and chemotherapy, provides a powerful tool that can be widely applied for the therapy of human diseases such as various cancers and genetic disorders.

## 4 Experimental Section

### Materials

Olaparib precursor (4‐(4‐fluoro‐3‐(piperazine‐1‐carbonyl)benzyl)phthalazin‐1(2H)‐one) was purchased from Ambeed. DBCO‐sulfo‐NHS (dibenzocyclooctyne‐sulfo‐N‐hydroxysuccinimidyl) ester was obtained from Click Chemistry Tools. Mal‐PEG_4_‐NHS (O‐[*N*‐(3‐maleimidopropionyl)aminoethyl]‐O′‐[3‐(*N*‐succinimidyloxy)−3‐oxopropyl]triethylene glycol) ester and low molecular weight branched polyethylenimine (Mw 2,000 Da) were purchased from Merck/MilliporeSigma. AzF (4‐azido‐l‐phenylalanine) was purchased from Chem‐Impex International. Sulfo‐succinimidyl‐4‐(*N*‐maleimidomethyl) cyclohexane‐1‐carboxylate (sulfo‐SMCC) and lipofectamine CRISPRMAX transfection reagent (CMAX) was purchased from Thermo Fisher Scientific. Primary antibodies mouse anti‐Rad52 (F‐7, sc‐365341), rabbit anti‐lamin A/C, and mouse anti‐transferrin; and horseradish peroxidase (HRP)‐conjugated secondary antibody were obtained from Santa Cruz Biotechnology. BODIPY‐FL‐PEG_4_‐DBCO was obtained from Jena Bioscience. TAT peptide (GRKKRRQRRRPQ) was obtained from GenScript. Primers used for DNA cloning were synthesized by Neoprobe (Table S1, Supporting Information). Primers used for target DNA preparation, sgRNA synthesis, and targeted deep sequencing were provided by Macrogen (Table S2, Supporting Information).

### Plasmids and Bacterial Strains

pETDuet SpCas9 wt His6 plasmid was constructed by inserting the *Streptococcus pyogenes* Cas9 gene with N‐terminal SV40 nuclear localization signal (NLS) sequence and C‐terminal His_6_‐tag into NcoI and AscI restriction sites. Additional silent mutations were inserted into the Cas9 gene to create NheI and EcoRI restriction sites. pRSFDuet AzFRS TyrT plasmid containing the AzFRS/tRNA_CUA_ pair developed by J. Chin et al. was used for amber codon suppression (Figure S22, Supporting Information for detailed plasmid maps).^[^ ^53^ ^]^ *E. coli* DH5α cells, and BL21 (DE3) competent cells were obtained from New England Biolabs. LB broth (Miller) was purchased from Ambrothia LLC.

### Expression and Purification of Cas9‐AzF Variants

For the construction of recombinant plasmids for expression of Cas9 with AzF incorporated at various positions, codons for Tyr or Phe were replaced with amber stop codons via overlap extension PCR (OE‐PCR). Recombinant plasmids for all target positions were introduced into competent *E. coli* DH5α cells via heat shock transformation. Bacterial colonies were selected from ampicillin‐containing (100 µg mL^−1^) LB agar plates and successful introduction of amber codons were confirmed by Sanger DNA sequencing. For protein expression, recombinant pETDuet SpCas9 wt His6 plasmid for each AzF incorporation site was transformed with the pRSFDuet AzFRS TyrT plasmid‐containing *E. coli* BL21 (DE3) competent cells. Bacterial cells were grown in LB medium supplemented with 100 µg mL^−1^ ampicillin and 50 µg mL^−1^ kanamycin and induced by the addition of 0.5 mM isopropyl β‐D‐1‐thiogalactopyranoside (IPTG) and 1 mM AzF at OD_600_ of 0.4–0.6. After incubation at 18 °C with gentle agitation for 32 h, cells were harvested and lysed by ultrasonication. Ni(II)‐NTA affinity chromatography was performed using ÄKTAprime Plus (GE Healthcare) and protein‐containing fractions were verified by sodium dodecyl‐sulfate polyacrylamide gel electrophoresis (SDS‐PAGE). Purified proteins were dialyzed against storage buffer (50 mM Tris‐Cl, 200 mM KCl, 0.1 mM EDTA, 20% glycerol, pH 8.0,) and concentrated with Amicon centrifugal filters (MWCO 30 kDa, Merck/MilliporeSigma).

### Synthesis of Cas9 Conjugates

To prepare Mal‐PEG_4_‐Olap, 5 mM Mal‐PEG_4_‐NHS ester was reacted with 15 mM olaparib precursor in dry acetonitrile for 2 h at room temperature. DBCO‐Olap was prepared by reacting 1 mM DBCO‐sulfo‐NHS with 10 mM olaparib precursor in PBS for 2 h at room temperature, in dark conditions. When the solution became cloudy, the supernatant was removed after centrifugation and the white precipitate was dissolved in dry acetonitrile. The synthesized products were analyzed by high‐performance liquid chromatography (FLC‐SHIMADZU LC‐2020). To synthesize Cas9‐Olap, Cas9‐AzF was reacted with either DBCO‐Olap or Mal‐PEG_4_‐Olap (molar ratio 1:100) in 50 mM Tris‐Cl, 200 mM KCl, and 0.1 mM EDTA for 24 h at 4 °C, and purified with an Amicon centrifugal filter (MWCO 30 kDa) to produce Cas9‐Olap1 or Cas9‐Olap2, respectively. For the synthesis of Cas9‐Olap3, Cas9‐Olap2 was further reacted with DBCO‐Olap, and purified as described above. For the synthesis of Cas9‐CP, sulfo‐SMCC or DBCO‐sulfo‐NHS was added to CP in 0.1 m sodium bicarbonate solution (pH 7.6) and reacted for 16 h at 4 °C (molar ratio 1:1). Dialysis (MWCO 500–1000 Da, Spectra/Por) was performed for 4 h against deionized water (DW), and the product was freeze‐dried. The products SMCC‐CP or DBCO‐CP were added to Cas9‐AzF in phosphate buffered saline (PBS, pH 7.2), reacted for 16 h at 4 °C, followed by dialysis (MWCO 100 kDa, Spectra/Por) for 8 h at 4 °C against storage buffer, to obtain Cas9‐CP2 or Cas9‐CP1, respectively. For the synthesis of Cas9‐Olap1‐CP2, Cas9‐CP2 was reacted with DBCO‐Olap (molar ratio 1:100) in 50 mM Tris‐Cl containing 200 mM KCl and 0.1 mM EDTA for 24 h at 4 °C, and purified with an amicon filter (30 kDa). For synthesis of Cas9‐Olap2‐CP1, Cas9‐Olap2 was reacted with DBCO‐CP, and purified as described for Cas9‐Olap1‐CP2. All products were characterized by SDS‐PAGE. For MALDI‐TOF spectrometry, the Cas9 conjugates were mixed with saturated sinapinic acid in 0.1% trifluoroacetic acid, 70% acetonitrile solution, and applied on an MTP 384 plate (Bruker Daltonics). After air drying until the white crystals were formed, analysis was performed using Bruker Daltonics AutoFlex maX in linear mode to obtain the cationic mass‐to‐charge ratio. Results were processed by Bruker Daltonics flexAnalysis 3.3 software.

### Physicochemical Characterization

The incorporation of AzF into Cas9 was qualitatively confirmed by reaction of Cas9‐AzF (100 µg) with BODIPY‐FL‐PEG_4_‐DBCO (molar ratio 1:10) for 2 h at room temperature, followed by PAGE and detecting fluorescence by UV irradiation using a gel imaging system. To quantify the number of azido groups incorporated into Cas9 or residual azido groups after conjugation, a fluorophore tagging method was performed. In brief, Cas9‐AzF or the conjugates (250 µg) were reacted with BODIPY‐FL‐PEG_4_‐DBCO (molar ratio 1:10) in PBS, followed by dialysis (MWCO 100 kDa; Spectra/Por) and the absorbance was measured using NanoDrop 2000 spectrophotometer (Thermo Fisher Scientific). For quantifying conjugation onto natural cysteine groups, the Cas9 conjugates (250 µg) were reacted with sulfo‐Cy7‐maleimide (molar ratio 1:20), followed by dialysis and measuring the absorbance using NanoDrop 2000. The hydrodynamic sizes and zeta potentials of the conjugate complexes were measured using ELSZ‐2000ZS (Otsuka). TEM was performed using JEM‐2100F (JEOL). AFM analysis was performed using NX10 (Park Systems).

### Synthesis of sgRNA and RNP Formation

sgRNA targeting the RAD52 gene was prepared using DNA templates consisting of a T7 promoter, crRNA, and tracrRNA, by PCR amplification with Power‐pfu (500 U µL^−1^) in Power‐pfu buffer (NanoHelix) added with dNTP (10 mM, Qiagen) and primers (50 pmol µL^−1^). Then, in vitro transcription was performed by adding the synthesized DNA templates, NTPs (100 mM, Jena Bioscience), to buffer including T7 RNA polymerase (50 000 units mL^−1^), 50 mM MgCl_2_, 0.1 M dithiothreitol, and RNase inhibitor murine (New England Biolabs), and incubating at 37 °C for 16 h. The synthesized sgRNA was purified with GeneAll Expin PCR SV kit (GeneAll Biotechnology), the concentration was measured using NanoDrop 2000, and the product was vacuum‐dried for 2 h using HyperVAC VC2124 (Hanil Scientific). RNPs were formed by adding Cas9‐AzF or Cas9 conjugates (500 nM) with sgRNA (500 nM) at a molar ratio of 1:1, and incubation at room temperature for 10 min.

### Endonuclease Activity

Cleavage activities of the purified Cas9‐AzF and conjugates were examined by adding PCR‐synthesized DNA including the RAD52 Exon 3 target locus (120 ng) with RNPs formed with the Cas9 conjugates (1 µg) and sgRNA (750 ng) specific for RAD52 Exon 3 in NEB3.1 buffer (25 mM Tris‐HCl, 50 mM NaCl, 5 mM MgCl_2_, and 50 µg mL^−1^ BSA, New England Biolabs). After incubation at 37 °C for 90 min, the resulting DNA fragments were analyzed by agarose gel electrophoresis, and cleavage efficiencies were determined by image analysis.

### Cell Culture

BRCA1‐deficient HCC1937 human breast cancer cells with the 5382C mutation and MDA‐MB‐231 human epithelial breast cancer cells were obtained from the Korean Cell Line Bank. Cells were cultured in RPMI‐1640 medium including 25 mM 4‐(2‐hydroxyethyl)−1‐piperazineethanesulfonic acid (HEPES) and l‐glutamine (Thermo Fisher), supplemented with 10% fetal bovine serum (FBS, GE Healthcare) and 1% penicillin/streptomycin (P/S, Thermo Fisher Scientific), in a CO_2_ incubator at 37 °C (BB15, Thermo Fisher).

### Cellular Uptake and Cytotoxicity Analyses

HCC1937 cells were seeded in 8‐well cell culture slides (SPL Life Sciences) at 5.0 × 10^4^ cells per well in RPMI‐1640 (10% FBS, 1% P/S). Cells were incubated for 24 h at 37 °C, washed with Opti‐MEM (10–20 mM HEPES, l‐glutamine, Thermo Fisher), and then treated with ComBiNE or control complexes including Cas9 labeled with Alexa Fluor 647 NHS ester (succinimidyl ester) (AF647, Thermo Fisher Scientific) at 500 nM Cas9 in Opti‐MEM (10–20 mM HEPES) for 4 h at 37 °C. Cells were then washed three times with Dulbecco's phosphate buffered saline (DPBS, Thermo Fisher), and fixed with 4% paraformaldehyde (Thermo Fisher) in DPBS for 10–20 min at RT. After washing twice with DPBS and permeabilization with 0.1% Triton‐X 100 (Merck/MilliporeSigma) in DPBS for 10 min, cells were again washed 1–2 times with DPBS, and stained with rhodamine‐phalloidin (ActinRed 555 ReadyProbes, Thermo Fisher Scientific) for 30 min. After washing, cells were mounted using Vectashield including 4′,6‐diamidino‐2‐phenylindole (DAPI) (Vector Laboratories), and observed using a laser scanning confocal microscope (LSM 880 or LSM 780, Carl Zeiss AG). All acquired images were processed using the ZEN Black and Blue Edition software (Carl Zeiss AG). To examine cytotoxicity, HCC1937 cells were treated with the formulations for 6–24 h, stained with Annexin V and PI (BD Biosciences) according to the manufacturer's instruction, and analyzed by flow cytometry (LSRFortessa, BD Biosciences).

### Anti‐Proliferation Assay

The anti‐proliferation of cells upon treatment of ComBiNE or control formulations was examined using the cell counting kit‐8 (CCK8, Dojindo Molecular Technologies) according to the manufacturer's instructions. In brief, HCC1937 or MDA‐MB‐231 cells were seeded in a 96‐well cell culture plate at a density of 5.0 × 10^3^ cells per well and incubated for 24 h for attachment. Cells were washed with culture media and treated with the formulations (500 nM Cas9) for 72 h at 37 °C. Then, 10 µL of the WST reagent was added, and incubated for 1 h at 37 °C, before measuring the absorbance at 450 nm using a microplate reader (Infinite M200 PRO, Tecan). Cell viability was determined based on normalization with values for the control (no treatment). All experiments were performed in triplicates for each treatment group.

### Examination of Gene Editing Efficiency

HCC1937 or MDA‐MB‐231 cells were seeded in 6‐ or 24‐well cell culture plates at 2.0 × 10^6^ or 5.0 × 10^4^ cells per well, respectively, in RPMI‐1640 (25 mM HEPES, l‐glutamine) containing 10% FBS and 1% P/S. After incubation for 24 h at 37 °C, cells were washed with fresh media, and then treated with the formulations (500 nM Cas9) for 48 h at 37 °C. The genomic DNA was isolated from the cells, and PCR was performed using Phusion high‐fidelity DNA polymerase (2 U µL^−1^, Thermo Fisher) in Phusion HF buffer added with dNTPs (10 mM), and primers (10 pmol µL^−1^). Products were purified using GeneAll Expin PCR SV kit and agarose gel electrophoresis, extracted from the gels, followed by targeted deep sequencing using MiniSeq (Illumina). Indel frequencies (%) were calculated using RGEN Cas‐analyzer (Seoul National University). For western blot analysis, the treated cells were washed with PBS, and total protein was extracted using the RIPA lysis and extraction buffer supplemented with protease and phosphatase inhibitors and quantified by the BCA assay (Thermo Fisher). SDS‐PAGE was performed by separation of 20 µg protein and blotted onto nitrocellulose using a Trans‐Blot turbo transfer system (Bio‐Rad Laboratories). Blocking solution (5% skim milk in 20 mM Tris, 150 mM NaCl, 0.1% Tween 20, pH 7.5, TBST) including primary antibody (1:1000) was added and incubated overnight at 4 °C, followed by incubation with HRP‐conjugated secondary antibody (1:3000) for 1 h at RT. After washing with TBST, enhanced chemiluminescence (ECL) substrate solution (Bio‐Rad Laboratories) was added according to the manufacturer's protocol, and the chemiluminescence was measured using the ChemiDoc imaging system (Bio‐Rad Laboratories).

### Apoptosis Assay

The apoptosis levels of cells treated with the ComBiNE and controls were assessed by Annexin V and propidium iodide (PI) staining (BD Biosciences) and flow cytometry, according to the manufacturer's instructions. In brief, HCC1937 cells were seeded in 6‐well cell culture plates at 1.0 × 10^6^ cells per well in RPMI‐1640 (10% FBS and 1% P/S), washed with media, and then treated with the formulation (500 nM Cas9) for 48 h at 37°C. Cells were then washed twice with cold PBS, pelleted by centrifugation, and resuspended in binding buffer containing 0.1 m HEPES/NaOH (pH 7.4), 1.4 M NaCl, and 25 mM CaCl_2_ at a concentration of 1.0 × 10^5^ cells. Then, cells were mixed with 5 µL FITC Annexin V and 5 µL PI, incubated for 15 min, followed by adding 400 µL of binding buffer and analysis using a FACS LSRFortessa (BD Biosciences). Data were processed by FACSDiva and FlowJo 10.8.1 software (BD Biosciences).

### Comet Assay

Cellular DNA damage was evaluated using the OxiSelect comet assay kit (Cell Biolabs) according to the manufacturer's instructions. In brief, HCC1937 cells were seeded in 6‐well cell culture plates at 5.0 × 10^5^ cells per well in RPMI‐1640 (10% FBS, 1% P/S), washed with fresh media after attachment, and treated with ComBiNE or control formulations (500 nM Cas9) in RPMI‐1640 (10% FBS, 1% P/S) for 48 h at 37 °C. Cells were then washed and resuspended in 1 ml of cold PBS at 1.0 × 10^5^ cells mL^−1^, and mixed with low melting‐point agarose at a 1:10 (v/v) ratio, followed by transferring 75 µl of the solution to agarose pre‐coated OxiSelect comet slides. After solidification of the agarose for 15 min at 4 °C, lysis buffer was treated for 30–60 min, and then immersed in an alkaline solution for 30 min at 4 °C. Electrophoresis was then carried out in TBE buffer, and cells were fixed with 70% ethanol for 5 min at RT, followed by air drying. Then, 100 µL Vista Green DNA dye (1:10 000) was added and incubated for 15 min at RT, followed by observation using a fluorescence microscope (LSM 780). The acquired images were processed using the ZEN Black and Blue Edition software. Cellular DNA damage was quantified by measuring the percentage of tail DNA (%comet‐positive cells with > 5% of DNA in the tail) and calculation using the formula *tail* *DNA*%  =  (*tail* *DNA* *intensity*/*cell* *DNA* *intensity*)  ×  100,  and *tail* *moment*  =  *tail* *DNA*%  ×  *tail* *length*, using Comet Score 2.0 imaging software (TriTrek).

### In Vivo Delivery and Efficacy

All animal studies were performed following authorized protocols and in accordance with the policies of the Institutional Animal Care and Use Committee (IACUC) of KAIST, with protocol no. KA2021‐103. HCC1937 xenograft tumors were generated by subcutaneous implantation of HCC1937 cells (2 × 10^6^ cells) suspended in 0.1 ml of PBS containing 50% (2:1 v/v) VitroGel hydrogel matrix (TheWell Bioscience) into the dorsal flank of female 5‐week‐old BALB/c athymic nude mice (Orient Bio). After confirming tumor growth 10 days after implantation, mice were divided into four treatment groups (*n* = 8): PBS (control), Cas9‐CP, ComBiNE (NT: non‐target), and ComBiNE, and treated intratumorally with the formulations (25 µg of Cas9 per injection) or PBS control 5 times every 3 days (day 0, 3, 6, 9, 12). The day of the first treatment was designated as day 0, and tumor growth was monitored using an electronic caliper and calculation based on the formula *tumor* *volume*  =  0.5 *LW* ^2^ (*L*  =  *length* *of* *the* *tumor*,  *W*  =  *width*) for 24 days. Mice body weights were measured each day for all mice. On day 5, two mice per group were sacrificed and tumor tissues were harvested for analyzing gene editing efficiencies (*n* = 2). All other mice were sacrificed on day 24 (*n* = 6 per group), and tumor tissues were harvested for gene editing analysis and functional assays including western blot, measuring apoptosis, and comet assay. To examine gene editing efficiencies, tumor tissues were minced, and genomic DNA was extracted using QIAamp fast DNA tissue kit (Qiagen). PCR was performed with genomic DNA (50 ng) for library preparation, followed by targeted deep sequencing analyses. For the western blot analysis, apoptosis assay, and comet assay, tumor tissues were minced into 1–3 mm^3^ pieces, followed by incubation with 1 mg mL^−1^ collagenase II and 100 Kunitz mL^−1^ DNase I (Merck/MilliporeSigma) for 1 h at 37 °C and 5% CO_2_ with gentle agitation. Western blot and comet assay were performed according to the description in the above section for in vitro analysis. For the apoptosis assay, single cells were obtained using cell strainers (70 µm and 40 µm, Corning), followed by Annexin V and PI staining and flow cytometry, as described above. All mice were housed in groups and maintained in a specific pathogen‐free environment.

### Statistical Analysis

All statistical data were expressed as mean ± standard deviation (SD). One‐way ANOVA followed by Tukey's or Dunnett's multiple comparison tests were performed to obtain the *p*‐value and determine significance, using GraphPad Prism 9. A value of *p* < 0.05 was considered statistically significant.

## Conflict of Interest

The authors declare no conflict of interest.

## Supporting information

Supporting Information

## Data Availability

The data that support the findings of this study are available from the corresponding author upon reasonable request.

## References

[1] O. Boutureira , G. J. Bernardes , Chem. Rev. 2015, 115, 2174. 25700113 10.1021/cr500399p

[2] E. A. Hoyt , P. M. Cal , B. L. Oliveira , G. J. Bernardes , Nat. Rev. Chem. 2019, 3, 147.

[3] L. Reguera , Y. Méndez , A. R. Humpierre , O. Valdés , D. G. Rivera , Acc. Chem. Res. 2018, 51, 1475. 29799718 10.1021/acs.accounts.8b00126

[4] A. Beck , L. Goetsch , C. Dumontet , N. Corvaïa , Nat. Rev. Drug Discovery 2017, 16, 315. 28303026 10.1038/nrd.2016.268

[5] S. J. Walsh , J. D. Bargh , F. M. Dannheim , A. R. Hanby , H. Seki , A. J. Counsell , X. Ou , E. Fowler , N. Ashman , Y. Takada , A. Isidro‐Llobet , Chem. Soc. Rev. 2021, 50, 1305. 33290462 10.1039/d0cs00310g

[6] S. A. Hurvitz , Nat Cancer 2022, 3, 1412. 36539503 10.1038/s43018-022-00495-7

[7] N. Krall , F. P. Da Cruz , O. Boutureira , G. J. Bernardes , Nat. Chem. 2016, 8, 103. 26791892 10.1038/nchem.2393

[8] P. Ochtrop , C. P. R. Hackenberger , Curr. Opin. Chem. Biol. 2020, 58, 28. 32645576 10.1016/j.cbpa.2020.04.017

[9] J. C. Jewett , C. R. Bertozzi , Chem. Soc. Rev. 2010, 39, 1272. 20349533 10.1039/b901970g PMC2865253

[10] S. L. Scinto , D. A. Bilodeau , R. Hincapie , W. Lee , S. S. Nguyen , M. Xu , C. W. Am Ende , M. G. Finn , K. Lang , Q. Lin , J. P. Pezacki , J. A. Prescher , M. S. Robillard , J. M. Fox , Nat. Rev. Methods Primers 2021, 1, 30. 34585143 10.1038/s43586-021-00028-z PMC8469592

[11] A. Yang , S. Ha , J. Ahn , R. Kim , S. Kim , Y. Lee , J. Kim , D. Söll , H. Y. Lee , H. S. Park , Science 2016, 354, 623. 27708052 10.1126/science.aah4428 PMC5135561

[12] K. Wu , N. A. Yee , S. Srinivasan , A. Mahmoodi , M. Zakharian , J. M. M. Oneto , M. Royzen , Chem. Sci. 2021, 12, 1259. 34163888 10.1039/d0sc06099b PMC8179178

[13] J. Ko , M. Wilkovitsch , J. Oh , R. H. Kohler , E. Bolli , M. J. Pittet , C. Vinegoni , D. B. Sykes , H. Mikula , R. Weissleder , J. C. Carlson , Nat. Biotechnol. 2022, 40, 1654. 35654978 10.1038/s41587-022-01339-6 PMC9669087

[14] J. Ko , K. Lucas , R. Kohler , E. A. Halabi , M. Wilkovitsch , J. C. Carlson , R. Weissleder , Adv. Sci. 2022, 9, 2200064. 10.1002/advs.202200064 PMC9405492 35750648

[15] M. Jinek , K. Chylinski , I. Fonfara , M. Hauer , J. A. Doudna , E. Charpentier , Science 2012, 337, 816. 22745249 10.1126/science.1225829 PMC6286148

[16] P. D. Hsu , E. S. Lander , F. Zhang , Cell 2014, 157, 1262. 24906146 10.1016/j.cell.2014.05.010 PMC4343198

[17] C. Liu , L. Zhang , H. Liu , K. Cheng , J. Controlled Release 2017, 266, 17. 10.1016/j.jconrel.2017.09.012 PMC5723556 28911805

[18] H. X. Wang , M. Li , C. M. Lee , S. Chakraborty , H. W. Kim , G. Bao , K. W. Leong , Chem. Rev. 2017, 117, 9874. 28640612 10.1021/acs.chemrev.6b00799

[19] H. Jin , L. Wang , R. Bernards , Nat. Rev. Drug Discovery 2022, 1. 10.1038/s41573-022-00615-z 36509911

[20] B. Al‐Lazikani , U. Banerji , P. Workman , Nat. Biotechnol. 2012, 30, 679. 22781697 10.1038/nbt.2284

[21] K. Bhatia , A. Das , Life Sci 2020, 258, 118134. 32717272 10.1016/j.lfs.2020.118134

[22] D. Zhang , G. Wang , X. Yu , T. Wei , L. Farbiak , L. T. Johnson , A. M. Taylor , J. Xu , Y. Hong , H. Zhu , D. J. Siegwart , Nat. Nanotechnol. 2022, 17, 777. 35551240 10.1038/s41565-022-01122-3 PMC9931497

[23] T. Wei , Q. Cheng , Y. L. Min , E. N. Olson , D. J. Siegwart , Nat. Commun. 2020, 11, 3232. 32591530 10.1038/s41467-020-17029-3 PMC7320157

[24] G. Chen , A. A. Abdeen , Y. Wang , P. K. Shahi , S. Robertson , R. Xie , M. Suzuki , B. R. Pattnaik , K. Saha , S. Gong , Nat. Nanotechnol. 2019, 14, 974. 31501532 10.1038/s41565-019-0539-2 PMC6778035

[25] D. N. Nguyen , T. L. Roth , P. J. Li , P. A. Chen , R. Apathy , M. R. Mamedov , L. T. Vo , V. R. Tobin , D. Goodman , E. Shifrut , J. A. Bluestone , Nat. Biotechnol. 2020, 38, 44. 31819258 10.1038/s41587-019-0325-6 PMC6954310

[26] K. Lee , M. Conboy , H. M. Park , F. Jiang , H. J. Kim , M. A. Dewitt , V. A. Mackley , K. Chang , A. Rao , C. Skinner , T. Shobha , Nat. Biomed. Eng. 2017, 1, 889. 29805845 10.1038/s41551-017-0137-2 PMC5968829

[27] H.‐X. Wang , Z. Song , Y.‐H. Lao , X. Xu , J. Gong , D. Cheng , S. Chakraborty , J. S. Park , M. Li , D. Huang , L. Yin , J. Cheng , K. W. Leong , Proc. Natl. Acad. Sci. USA 2018, 115, 4903. 29686087 10.1073/pnas.1712963115 PMC5948953

[28] S. M. Kim , S. C. Shin , E. E. Kim , S.‐H. Kim , K. Park , S. J. Oh , M. Jang , ACS Nano 2018, 12, 7750. 30028587 10.1021/acsnano.8b01670

[29] H. Tang , X. Xu , Y. Chen , H. Xin , T. Wan , B. Li , H. Pan , D. Li , Y. Ping , Adv. Mater. 2021, 33, 200603. 10.1002/adma.202006003 33538047

[30] J. Lee , Y. K. Kang , E. Oh , J. Jeong , S. H. Im , D. K. Kim , H. Lee , S. G. Kim , K. Jung , H. J. Chung , Chem. Mater. 2022, 34, 547.

[31] R. Rouet , B. A. Thuma , M. D. Roy , N. G. Lintner , D. M. Rubitski , J. E. Finley , H. M. Wisniewska , R. Mendonsa , A. Hirsh , L. de Onate , J. Compte Barrón , T. F. McLellan , J. Bellenger , X. Feng , A. Varghese , B. A. Chrunyk , K. Borzilleri , K. D. Hesp , K. Zhou , N. Ma , M. Tu , R. Dullea , K. F. McClure , R. C. Wilson , S. Liras , V. Mascitti , J. A. Doudna , J. Am. Chem. Soc. 2018, 140, 6596. 29668265 10.1021/jacs.8b01551 PMC6002863

[32] X. Ling , B. Xie , X. Gao , L. Chang , W. Zheng , H. Chen , Y. Huang , L. Tan , M. Li , T. Liu , Sci. Adv. 2020, 6, eaaz0051. 32494588 10.1126/sciadv.aaz0051 PMC7250679

[33] Y. K. Kang , J. Lee , J. H. Lee , J. Jeong , D. K. Kim , S. Y. Yang , K. Jung , S. G. Kim , H. J. Chung , J Ind Eng Chem 2021, 102, 241.

[34] Y. K. Kang , K. Kwon , J. S. Ryu , H. N. Lee , C. Park , H. J. Chung , Bioconj. Chem. 2017, 28, 957. 10.1021/acs.bioconjchem.6b00676 28215090

[35] S. Ramakrishna , A. B. K. Dad , J. Beloor , R. Gopalappa , S. K. Lee , H. Kim , Genome Res. 2014, 24, 1020. 24696462 10.1101/gr.171264.113 PMC4032848

[36] X. Ling , X. Gao , L. Chang , H. Chen , X. Shi , T. Liu , Chem. Commun. 2020, 56, 7515. 10.1039/d0cc01432j 32510061

[37] X. Ling , L. Chang , H. Chen , X. Gao , J. Yin , Y. Zuo , Y. Huang , B. Zhang , J. Hu , T. Liu , Mol. Cell 2021, 81, 4747. 34648747 10.1016/j.molcel.2021.09.021

[38] H. Nishimasu , F. A. Ran , P. D. Hsu , S. Konermann , S. I. Shehata , N. Dohmae , R. Ishitani , F. Zhang , O. Nureki , Cell 2014, 156, 935. 24529477 10.1016/j.cell.2014.02.001 PMC4139937

[39] C. Huai , G. Li , R. Yao , Y. Zhang , M. Cao , L. Kong , C. Jia , H. Yuan , H. Chen , D. Lu , Q. Huang , Nat. Commun. 2017, 8, 1375. 29123204 10.1038/s41467-017-01496-2 PMC5680257

[40] C. J. Lord , A. Ashworth , Science 2017, 355, 1152. 28302823 10.1126/science.aam7344 PMC6175050

[41] S. Kummar , A. Chen , R. E. Parchment , R. J. Kinders , J. Ji , J. E. Tomaszewski , J. H. Doroshow , BMC Med. 2012, 10, 25. 22401667 10.1186/1741-7015-10-25 PMC3312820

[42] W. Sun , W. Ki , J. M. Hall , Q. Hu , C. Wang , C. L. Beisel , Z. Gu , Angew. Chem. 2015, 127, 12197. 10.1002/anie.201506030 PMC4677991 26310292

[43] J. Nam , S. Son , K. S. Park , J. J. Moon , Adv. Sci. 2021, 8, 2002577. 10.1002/advs.202002577 PMC7927624 33717838

[44] H. Bloomer , J. Khirallah , Y. Li , Q. Xu , Adv. Drug Delivery Rev. 2022, 181, 114087. 10.1016/j.addr.2021.114087 PMC8844242 34942274

[45] K. Sullivan‐Reed , E. Bolton‐Gillespie , Y. Dasgupta , S. Langer , M. Siciliano , M. Nieborowska‐Skorska , K. Hanamshet , E. A. Belyaeva , A. J. Bernhardy , J. Lee , M. Moore , Cell Rep. 2018, 23, 3127. 29898385 10.1016/j.celrep.2018.05.034 PMC6082171

[46] A. Huang , L. A. Garraway , A. Ashworth , B. Weber , Nat. Rev. Drug Discovery 2020, 19, 23. 31712683 10.1038/s41573-019-0046-z

[47] G. Guidotti , L. Brambilla , D. Rossi , Trends Pharmacol. Sci. 2017, 38, 406. 28209404 10.1016/j.tips.2017.01.003

[48] C. J. Lord , A. N. Tutt , A. Ashworth , Annu Rev Med 2015, 66, 455. 25341009 10.1146/annurev-med-050913-022545

[49] M. A. Tyumentseva , A. I. Tyumentsev , V. G. Akimkin , PLoS One 2021, 16, 0259812. 10.1371/journal.pone.0259812 PMC8577758 34752487

[50] A. Ghaemi , E. Bagheri , K. Abnous , S. M. Taghdisi , M. Ramezani , M. Alibolandi , Life Sci. 2021, 267, 118969. 33385410 10.1016/j.lfs.2020.118969

[51] X.‐Z. Chen , R. Guo , C. Zhao , J. Xu , H. Song , H. Yu , C. Pilarsky , F. Nainu , J.‐Q. Li , X.‐K. Zhou , K.‐Y. Zhang , Front Pharmacol 2022, 13, 939090. 35935840 10.3389/fphar.2022.939090 PMC9353945

[52] Z. Zhao , A. Anselmo , S. Mitragotri , Bioeng. Transl. Med. 2022, 7, 10258. 10.1002/btm2.10258 PMC8780015 35079633

[53] J. W. Chin , S. W. Santoro , A. B. Martin , D. S. King , L. Wang , P. G. Schultz , J. Am. Chem. Soc. 2002, 124, 9026. 12148987 10.1021/ja027007w

