# Supplementary material for: Bioorthogonal CRISPR/Cas9‐Drug Conjugate: A Combinatorial Nanomedicine Platform
Source: Adv Sci (Weinh). 2023 Jul 23;10(27):2302253. doi: 10.1002/advs.202302253 (PMC10520654; doi:10.1002/advs.202302253)
Supplement: Supplementary file 1 — Supporting Information [file ADVS-10-2302253-s001.pdf]

ADVANCED SCIENCE
Open Access

Supporting Information

for *Adv. Sci.*, DOI 10.1002/advs.202302253

Bioorthogonal CRISPR/Cas9-Drug Conjugate: A Combinatorial Nanomedicine Platform

Marcel Janis Beha, Joo-Chan Kim, San Hae Im, Yunsu Kim, Seungju Yang, Juhee Lee, Yu Ri Nam, Haeshin Lee, Hee-Sung Park and Hyun Jung Chung**

Supporting Information

Bioorthogonal CRISPR/Cas9-Drug conjugate: A Combinatorial Nanomedicine Platform

Marcel Janis Beha,† Joo-Chan Kim,† San Hae Im,† Yunsu Kim, Seungju Yang, Juhee Lee, Yu Ri Nam, Haeshin Lee, Hee-Sung Park, and Hyun Jung Chung**

M. J. Beha, S. H. Im, S. J. Yang, J. H. Lee, H. J. Chung
Department of Biological Sciences, Korea Advanced Institute of Science and Technology, Daejeon 34141, Republic of Korea
Email: hyunjc@kaist.ac.kr

J. C. Kim, Y. S. Kim, Y. R. Nam, H. Lee, H. S. Park
Department of Chemistry, Korea Advanced Institute of Science and Technology, Daejeon 34141, Republic of Korea
Email: hspark@kaist.ac.kr

H. J. Chung
Graduate School of Nanoscience and Technology, Korea Advanced Institute of Science and Technology, Daejeon 34141, Republic of Korea
Email: hyunjc@kaist.ac.kr

† These authors contributed equally: Marcel Janis Beha, Joo-Chan Kim, San Hae Im.

Table of Contents

Supporting Methods ……………………………………………………………...……………..2
Supporting Tables ……………………………………………………………...………..5
Supporting Figures ………………………………………………………………..……..7

Supporting Methods

Sequence for Cas9 plasmid:

N-terminal HA – SV40 NLS – TEV site – SpCas9 – His6 C-terminal

Nucleotide sequence (4212 bp)

5’-

ATGGTGTACCCCTACGACGTGCCCGACTACGCCGAATTGCCTCCAAAAAAGAAGAGAAAGGTAGGGATCGAGAACCTGTACT

T

CCAGGGCGACAAGAAGTACAGCATCGGCCTGGACATCGGTACCAACAGCGTGGGCTGGGCCGTGATCACCGACGAGTACAA

GGTGCCCAGCAAGAAGTTCAAGGTGCTGGGCAACACCGACCGCCACAGCATCAAGAAGAACCTGATCGGCGCCCTGCTGTTC

GACAGCGGCGAGACCGCCGAGGCCACCCGCCTGAAGCGCACCGCCCGCCGCCGCTACACCCGCCGCAAGAACCGCATCTGCT

ACCTGCAGGAGATCTTCAGCAACGAGATGGCCAAGGTGGACGACAGCTTCTTCCACCGCCTGGAGGAGAGCTTCCTGGTGGA

GGAGGACAAGAAGCACGAGCGCCACCCCATCTTCGGCAACATCGTGGACGAGGTGGCCTACCACGAGAAGTACCCCACCATC

TACCACCTGCGCAAGAAGCTGGTGGACAGCACCGACAAGGCCGACCTGCGCCTGATCTACCTGGCCCTGGCCCACATGATCA

AGTTCCGCGGCCACTTCCTGATCGAGGGCGACCTGAACCCCGACAACAGCGACGTGGACAAGCTGTTCATCCAGCTGGTGCA

GACCTACAACCAGCTGTTCGAGGAGAACCCCATCAACGCCAGCGGCGTGGACGCCAAGGCCATCCTGAGCGCCCGCCTGAGC

AAGAGCCGCCGCCTGGAGAACCTGATCGCCCAGCTGCCCGGCGAGAAGAAGAACGGCCTGTTCGGCAACCTGATCGCCCTGA

GCCTGGGCCTGACCCCCAACTTCAAGAGCAACTTCGACCTGGCCGAGGACGCCAAGCTGCAGCTGAGCAAGGACACCTACGA

CGACGACCTGGACAACCTGCTGGCCCAGATCGGCGACCAGTACGCCGACCTGTTCCTGGCCGCCAAGAACCTGAGCGACGCC

ATCCTGCTGAGCGACATCCTGCGCGTGAACACCGAGATCACCAAGGCCCCCCTGAGCGCCAGCATGATCAAGCGCTACGACG

AGCACCACCAGGACCTGACCCTGCTGAAGGCCCTGGTGCGCCAGCAGCTGCCCGAGAAGTACAAGGAGATCTTCTTCGACCA

GAGCAAGAACGGCTACGCCGGCTACATCGACGGCGGCGCtAGCCAGGAGGAGTTCTACAAGTTCATCAAGCCCATCCTGGAG

AAGATGGACGGCACCGAGGAGCTGCTGGTGAAGCTGAACCGCGAGGACCTGCTGCGCAAGCAGCGCACCTTCGACAACGGC

AGCATCCCCCACCAGATCCACCTGGGCGAGCTGCACGCCATCCTGCGCCGCCAGGAGGACTTCTACCCCTTCCTGAAGGACA

ACCGCGAGAAGATCGAGAAGATCCTGACCTTCCGCATCCCCTACTACGTGGGCCCCCTGGCCCGCGGCAACAGCCGCTTCGC

CTGGATGACCCGCAAGAGCGAGGAGACCATCACCCCCTGGAACTTCGAGGAGGTGGTGGACAAGGGCGCCAGCGCCCAGAG

CTTCATCGAGCGCATGACCAACTTCGACAAGAACCTGCCCAACGAGAAGGTGCTGCCCAAGCACAGCCTGCTGTACGAGTAC

TTCACCGTGTACAACGAGCTGACCAAGGTGAAGTACGTGACCGAGGGCATGCGCAAGCCCGCCTTCCTGAGCGGCGAGCAGA

AGAAGGCCATCGTGGACCTGCTGTTCAAGACCAACCGCAAGGTGACCGTGAAGCAGCTGAAGGAGGACTACTTCAAGAAGA

TCGAGTGCTTCGACAGCGTGGAGATCAGCGGCGTGGAGGACCGCTTCAACGCCAGCCTGGGCACCTACCACGACCTGCTGAA

GATCATCAAGGACAAGGACTTCCTGGACAACGAGGAGAACGAGGACATCCTGGAGGACATCGTGCTGACCCTGACCCTGTTC

GAGGACCGCGAGATGATCGAGGAGCGCCTGAAGACCTACGCCCACCTGTTCGACGACAAGGTGATGAAGCAGCTGAAGCGC

CGCCGCTACACCGGCTGGGGCCGCCTGAGCCGCAAGCTTATCAACGGCATCCGCGACAAGCAGAGCGGCAAGACCATCCTGG

ACTTCCTGAAGAGCGACGGCTTCGCCAACCGCAACTTCATGCAGCTGATCCACGACGACAGCCTGACCTTCAAGGAGGACAT

CCAGAAGGCCCAGGTGAGCGGCCAGGGCGACAGCCTGCACGAGCACATCGCCAACCTGGCCGGCAGCCCCGCCATCAAGAA

GGGCATCCTGCAGACCGTGAAGGTGGTGGACGAGCTGGTGAAGGTGATGGGCCGCCACAAGCCCGAGAACATCGTGATCGA

GATGGCCCGCGAGAACCAGACCACCCAGAAGGGCCAGAAGAACAGCCGCGAGCGCATGAAGCGCATCGAGGAGGGCATCA

AGGAGCTGGGCAGCCAGATCCTGAAGGAGCACCCCGTGGAGAACACCCAGCTGCAGAACGAGAAGCTGTACCTGTACTACCT

GCAGAACGGCCGCGACATGTACGTGGACCAGGAGCTGGACATCAACCGCCTGAGCGACTACGACGTGGACCACATCGTGCCC

CAGAGCTTCCTGAAGGACGACAGCATCGACAACAAGGTGCTGACCCGCAGCGACAAGAACCGCGGCAAGAGCGACAACGTG

CCCAGCGAGGAGGTGGTGAAGAAGATGAAGAACTACTGGCGCCAGCTGCTGAACGCCAAGCTGATCACCCAGCGCAAGTTC

GACAACCTGACCAAGGCCGAGCGCGGCGGCCTGAGCGAGCTGGACAAGGCCGGCTTCATCAAGCGCCAGCTGGTGGAGACC

CGCCAGATCACCAAGCACGTGGCCCAGATCCTGGACAGCCGCATGAACACCAAGTACGACGAGAACGACAAGCTGATCCGC

GAGGTGAAGGTGATCACCCTGAAGAGCAAGCTGGTGAGCGACTTCCGCAAGGACTTCCAGTTCTACAAGGTGCGCGAGATCA

ACAACTACCACCACGCCCACGACGCCTACCTGAACGCCGTGGTGGGCACCGCCCTGATCAAGAAGTACCCCAAGCTGGAGAG

CGAATTCGTGTACGGCGACTACAAGGTGTACGACGTGCGCAAGATGATCGCCAAGAGCGAGCAGGAGATCGGCAAGGCCAC

CGCCAAGTACTTCTTCTACAGCAACATCATGAACTTCTTCAAGACCGAGATCACCCTGGCCAACGGCGAGATCCGCAAGCGCC

CCCTGATCGAGACCAACGGCGAGACCGGCGAGATCGTGTGGGACAAGGGCCGCGACTTCGCCACCGTGCGCAAGGTGCTGA

GCATGCCCCAGGTGAACATCGTGAAGAAGACCGAGGTGCAGACCGGCGGCTTCAGCAAGGAGAGCATCCTGCCCAAGCGCA

ACAGCGACAAGCTGATCGCCCGCAAGAAGGACTGGGACCCCAAGAAGTACGGCGGCTTCGACAGCCCCACCGTGGCCTACA

GCGTGCTGGTGGTGGCCAAGGTGGAGAAGGGCAAGAGCAAGAAGCTGAAGAGCGTGAAGGAGCTGCTGGGCATCACCATCA

TGGAGCGCAGCAGCTTCGAGAAGAACCCCATCGACTTCCTGGAGGCCAAGGGCTACAAGGAGGTGAAGAAGGACCTGATCA

TCAAGCTGCCCAAGTACAGCCTGTTCGAGCTGGAGAACGGCCGCAAGCGCATGCTGGCCAGCGCCGGCGAGCTGCAGAAGG

GCAACGAGCTGGCCCTGCCCAGCAAGTACGTGAACTTCCTGTACCTGGCCAGCCACTACGAGAAGCTGAAGGGCAGCCCCGA

GGACAACGAGCAGAAGCAGCTGTTCGTGGAGCAGCACAAGCACTACCTGGACGAGATCATCGAGCAGATCAGCGAGTTCAG

CAAGCGCGTGATCCTGGCCGACGCCAACCTGGACAAGGTGCTGAGCGCCTACAACAAGCACCGCGACAAGCCCATCCGCGAG

CAGGCCGAGAACATCATCCACCTGTTCACCCTGACCAACCTGGGCGCCCCCGCCGCCTTCAAGTACTTCGACACCACCATCGA

CCGCAAGCGCTACACCAGCACCAAGGAGGTGCTGGACGCCACCCTGATCCACCAGAGCATCACCGGTCTGTACGAGACCCGC

ATCGACCTGAGCCAGCTGGGTGGCGACCACCACCATCATCACCATTAA-3’

Amino acid sequence:

MVYPYDVPDYAELPPKKKRKVGIENLYFQGDKKYSIGLDIGTNSVGWAVITDEYKVPSKKFKVLGNTDRHSIKKNLIGALLFDSGE

TAEATRLKRTARRRYTRRKNRICYLQEIFSNEMAKVDDSFFHRLEESFLVEEDKKHERHPIFGNIVDEVAYHEKYPTIYHLRKKLVD

STDKADLRLIYLALAHMIKFRGHFLIEGDLNPDNSDVDKLFIQLVQTYNQLFEENPINASGVDAKAILSARLSKSRRLENLIAQLPGE

KKNGLFGNLIALSLGLTPNFKSNFDLAEDAKLQLSKDTYDDDLDNLLAQIGDQYADLFLAAKNLSDAILLSDILRVNTEITKAPLSA

SMIKRYDEHHQDLTLLKALVRQQLPEKYKEIFFDQSKNGYAGYIDGGASQEEFYKFIKPILEKMDGTEELLVKLNREDLLRKQRTF

DNGSIPHQIHLGELHAILRRQEDFYPFLKDNREKIEKILTFRIPYYVGPLARGNSRFAWMTRKSEETITPWNFEEVVDKGASAQSFIER

MTNFDKNLPNEKVLPKHSLLYEYFTVYNELTKVKYVTEGMRKPAFLSGEQKKAIVDLLFKTNRKVTVKQLKEDYFKKIECFDSVEI

SGVEDRFNASLGTYHDLLKIIKDKDFLDNEENEDILEDIVLTLTLFEDREMIEERLKTYAHLFDDKVMKQLKRRRYTGWGRLSRKLI

NGIRDKQSGKTILDFLKSDGFANRNFMQLIHDDSLTFKEDIQKAQVSGQGDSLHEHIANLAGSPAIKKGILQTVKVVDELVKVMGR

HKPENIVIEMARENQTTQKGQKNSRERMKRIEEGIKELGSQILKEHPVENTQLQNEKLYLYYLQNGRDMYVDQELDINRLSDYDVD

HIVPQSFLKDDSIDNKVLTRSDKNRGKSDNVPSEEVVKKMKNYWRQLLNAKLITQRKFDNLTKAERGGLSELDKAGFIKRQLVET

RQITKHVAQILDSRMNTKYDENDKLIREVKVITLKSKLVSDFRKDFQFYKVREINNYHHAHDAYLNAVVGTALIKKYPKLESEFVY

GDYKVYDVRKMIAKSEQEIGKATAKYFFYSNIMNFFKTEITLANGEIRKRPLIETNGETGEIVWDKGRDFATVRKVLSMPQVNIVK

KTEVQTGGFSKESILPKRNSDKLIARKKDWDPKKYGGFDSPTVAYSVLVVAKVEKGKSKKLKSVKELLGITIMERSSFEKNPIDFLE

AKGYKEVKKDLIIKLPKYSLFELENGRKRMLASAGELQKGNELALPSKYVNFLYLASHYEKLKGSPEDNEQKQLFVEQHKHYLDEI

IEQISEFSKRVILADANLDKVLSAYNKHRDKPIREQAENIIHLFTLTNLGAPAAFKYFDTTIDRKRYTSTKEVLDATLIHQSITGLYETR

IDLSQLGGDHHHHHH

Supporting Tables

Table S1. Primer sequences used for DNA cloning of plasmids for Cas9-AzF expression.

Name of plasmid/primer	Length (bp)	Sequences
**Duet_forward**	19	5'-GGGATCTCGACGCTCTCCC-3'
**Duet_reverse**	19	5'-GCTAGTTATTGCTCAGCGG-3'
**SpCas9_HindIII_forward**	22	5'-CCTGAGCCGCAAGCTTATCAAC-3'
**SpCas9_HindIII_reverse**	21	5'-TTGATAAGCTTGCGGCTCAGG-3'
**SpCas9_NheI_for**	37	5'-GCGCCAGCCAGGAGGAGTTCTACAAGTTCATCAAGCC-3'
**SpCas9_NheI_rev**	39	5’-GTAGAACTCCTCCTGGCTGGCGCCGCCGTCGATGTAGCC-3’
**SpCas9_EcoRI_for**	37	5’-CCCCAAGCTGGAGAGCGAATTCGTGTACGGCGACTAC-3’
**SpCas9_EcoRI_rev**	37	5’-GTAGTCGCCGTACACGAATTCGCTCTCCAGCTTGGGG-3’
**SpCas9_NcoI_HA_forward**	33	5'-TTTTCCATGGTGTACCCCTACGACGTGCCCGAC-3'
**Y5amber_forward**	21	5'-TAGAGCATCGGCCTGGACATC-3'
**Y5amber_reverse**	40	5'-GATGTCCAGGCCGATGCTCTACTTCTTGTCGCCCTGGAAG-3'
**Y81amber_forward**	21	5’-TAGCTGCAGGAGATCTTCAGC-3’
**Y81amber_reverse**	39	5’-GCTGAAGATCTCCTGCAGCTAGCAGATGCGGTTCTTGCG-3'
**Y192amber_forward**	38	5’-ATCCAGCTGGTGCAGACCTACAACCAGCTGTTCGAGGA-3’
**Y192amber_reverse**	39	5’-CTCCTCGAACAGCTGGTTGTAGGTCTGCACCAGCTGGAT-3'
**Y286amber_forward**	38	5'-GCCCAGATCGGCGACCAGTAGGCCGACCTGTTCCTGGC-3'
**Y286amber_reverse**	21	5'-CTACTGGTCGCCGATCTGGGC-3'
**F375amber_forward**	39	5'-CAGGAGGAGTTCTACAAGTAGATCAAGCCCATCCTGGAG-3'
**F375amber_reverse**	21	5'-CTACTTGTAGAACTCCTCCTG-3'
**Y430amber_forward**	42	5’-CTGCGCCGCCAGGAGGACTTCTAGCCCTTCCTGAAGGACAAC-3’
**Y430amber_reverse**	24	5’-CTAGAAGTCCTCCTGGCGGCGCAG-3'
**Y639amber_forward**	39	5’-GAGGAGCGCCTGAAGACCTAGGCCCACCTGTTCGACGAC-3’
**Y639amber_reverse**	21	5’-CTAGGTCTTCAGGCGCTCCTC-3'
**F688amber_forward**	21	5’-TAGGCCACCCGCAACTTCATG-3’
**F688amber_reverse**	40	5’-CATGAAGTTGCGGTTGGCCTAGCCGTCGCTCTTCAGGAAG-3'
**Y836amber_forward**	21	5’-TAGGACGTGGACCACATCGTG-3’
**Y836amber_reverse**	40	5’-CACGATGTGGTCCACGTCCTAGTCGCTCAGGCGGTTGATG-3'
**Y943amber_forward**	23	5’-CAAGTAGGACGAGAACGACAAGC-3’
**Y943amber_reverse**	40	5’-CAGCTTGTCGTTCTCGTCCTACTTGGTGTTCATGCGGCTG-3'
**F1235amber_forward**	37	5’-CCCAGCAAGTACGTGAACTAGCTGTACCTGGCCAGCC-3’
**F1235amber_reverse**	21	5’-CTAGTTCACGTACTTGCTGGG-3'
**Y1326amber_forward**	39	5’-GCCCCCGCCGCCTTCAAGTAGTTCGACACCACCATCGAC-3’
**Y1326amber_reverse**	24	5’-CTACTTGAAGGCGGCGGGGGCGCC-3'
**Y1336amber_forward**	39	5’-ACCATCGACCGCAAGCGCTAGACCAGCACCAAGGAGGTG-3’
**Y1336amber_reverse**	22	5’-CTAGCGCTTGCGGTCGATGGTG-3'
**Y1356amber_forward**	39	5’-CAGAGCATCACCGGTCTGTAGGAGACCCGCATCGACCTG-3’
**Y1356amber_reverse**	21	5’-CTACAGACCGGTGATGCTCTG-3'

Table S2. Sequences of primers used for target DNA and sgRNA synthesis, and targeted deep sequencing.

Name	Length (bp)	Sequences
**RAD52 Exon3 target DNA (forward)**	20	5'-GGAAAGGACAAATGGAAGCA-3'
**RAD52 Exon3 target DNA (reverse)**	20	5'-CGGTTATGCGAGTGTCAAGA-3'
**RAD52 Exon3 sgRNA (forward)**	85	5'-GAAATTAATACGACTCACTATAGAGAATACATAAGTAG CCGCAGTTTTAGAGCTAGAAATAGCAAGTTAAAATAAGG CTAGTCCG-3'
**Tracr sgRNA (reverse)**	64	5'-AAAAAAGCACCGACTCGGTGCCACTTTTTCAA GTTGATAACGGACTAGCCTTATTTTAACTTGC-3'
**RAD52 Exon3 NGS primer-1 (forward)**	20	5'-GGAAAGGACAAATGGAAGCA-3'
**RAD52 Exon3 NGS primer-1 (reverse)**	20	5'-CGGTTATGCGAGTGTCAAGA-3'
**RAD52 Exon3 NGS primer-2 (forward)**	21	5'-TGCCAGTACACAGCAGAAGAG-3'
**RAD52 Exon3 NGS primer-2 (reverse)**	20	5'-CATCGGAGTCTGCGGTATGG-3'

Supporting Figures

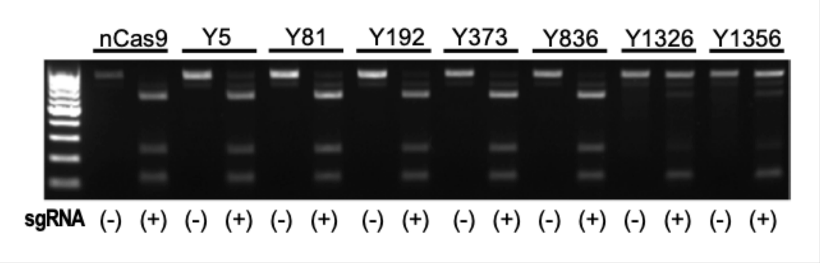

Figure S1. Cleavage activity of Cas9-AzF variants, generated by AzF incorporation into various positions. Cas9-AzF was complexed with RAD52 sgRNA (molar ratio 1:1), and treated to RAD52 target DNA.

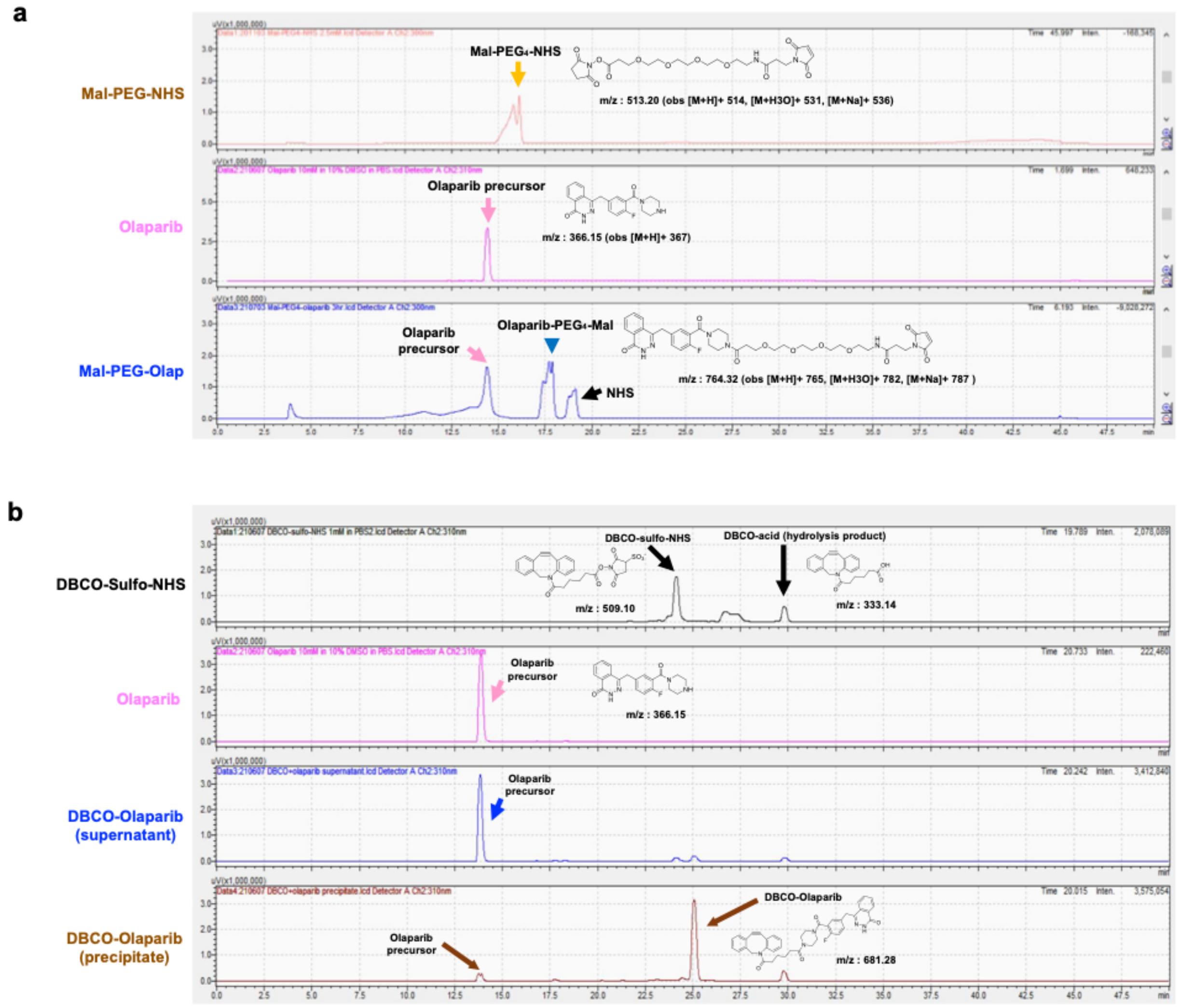

Figure S2. Analyses of functionalized olaparib by HPLC-MS. (a) Mal-PEG4-NHS ester or (b) DBCO-sulfo-NHS ester was reacted with olaparib (molar ratio 1:10), followed by HPLC purification.

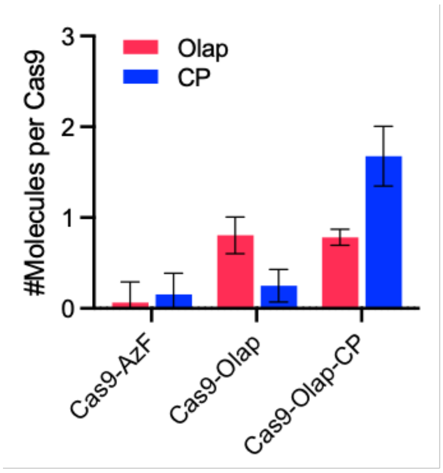

Figure S3. Conjugation efficiencies of olaparib and CP reacted with Cas9-AzF. Residual azido and sulfhydryl groups were measured by a fluorescence tagging method.

a

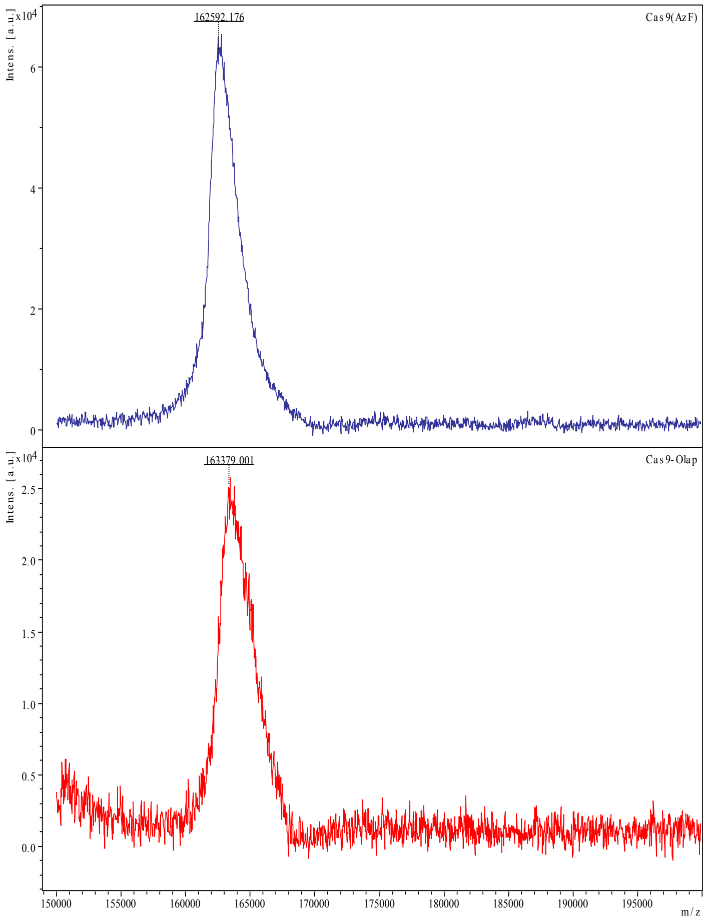

b

Protein	Theoretical m/z	Experimental m/z
Cas9-AzF	162598.43	162592.18
Cas9-Olap	163279.71	163379.00

Figure S4. Characterization of Cas9 conjugates by MALDI-TOF. (a) Mass spectra of Cas9-Olap and unmodified Cas9-AzF. (b) Theoretical and experimental values of m/z ratios.

a

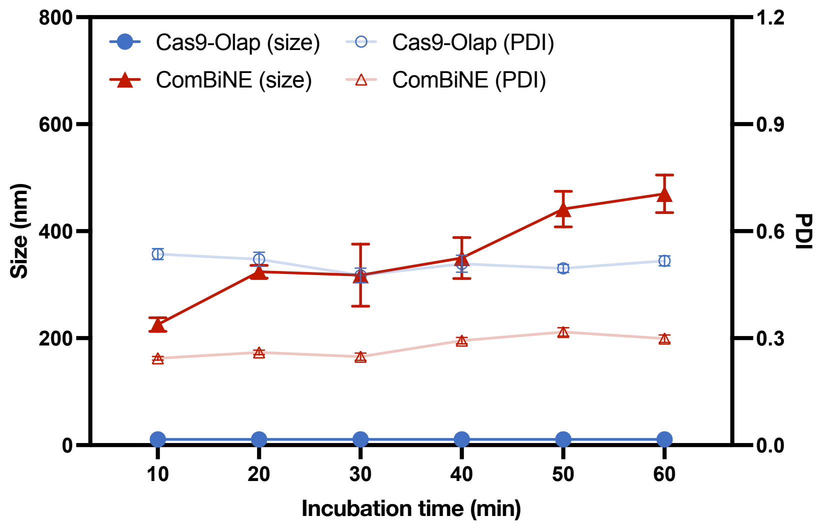

b

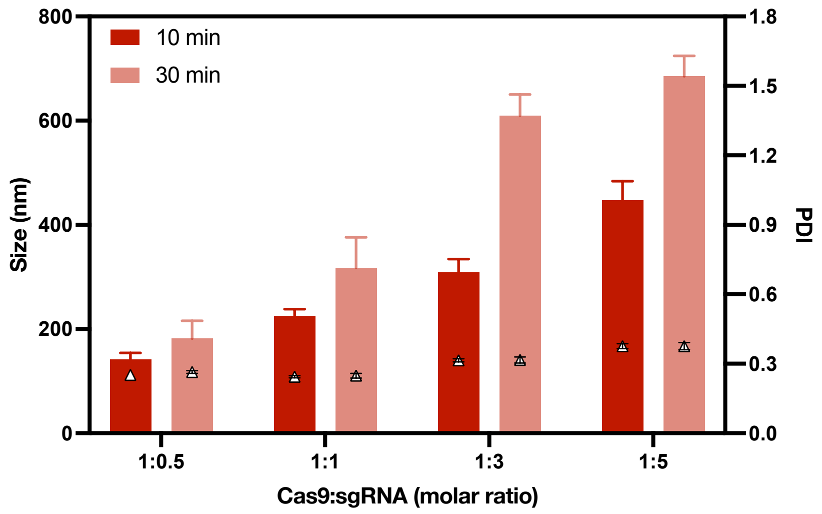

Figure S5. Hydrodynamic sizes and PDI values of ComBiNE according to various conditions: (a) ComBiNE and control (Cas9-Olap) at various incubation times after complexation (Cas9:sgRNA molar ratio 1:1). (b) ComBiNE at various Cas9:sgRNA molar ratios.

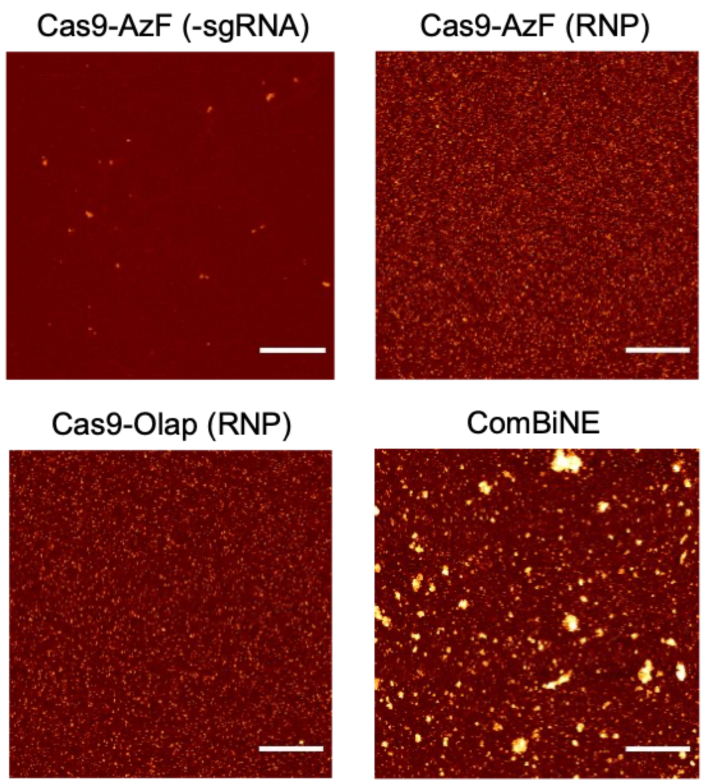

Figure S6. 2D AFM images of ComBiNE and control formulations. Cas9-AzF variant for Y373 was conjugated with olaparib and CP, and complexed with sgRNA (1:1). Scale bar 200 nm.

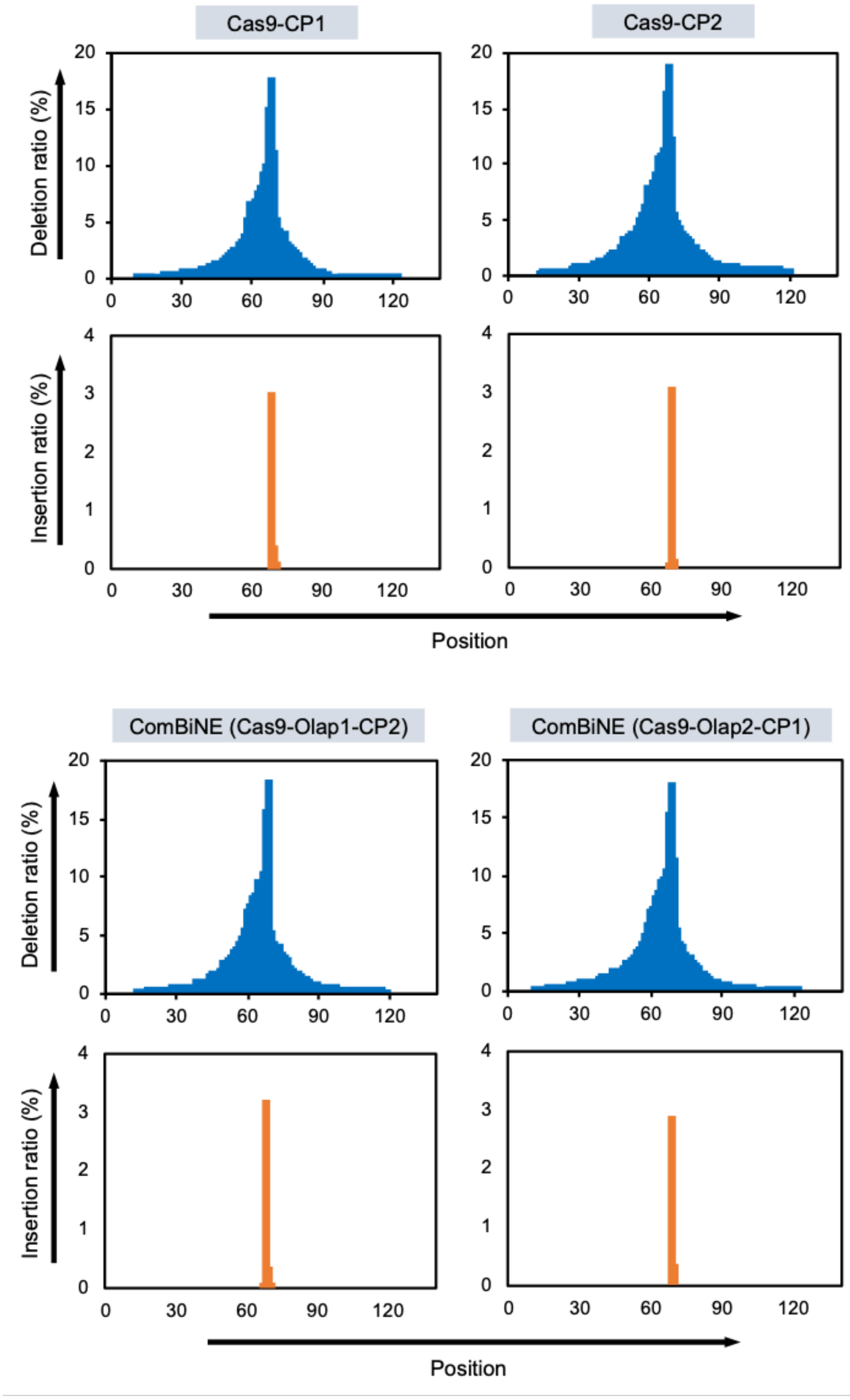

Figure S7. Gene editing patterns by treatment of ComBiNE. Deletion and insertion ratios (%) in the RAD52 gene obtained by targeted deep sequencing of HCC1937 cells, after treatment with ComBiNE and Cas9-CP RNPs targeting RAD52 for 48 h at 500 nM.

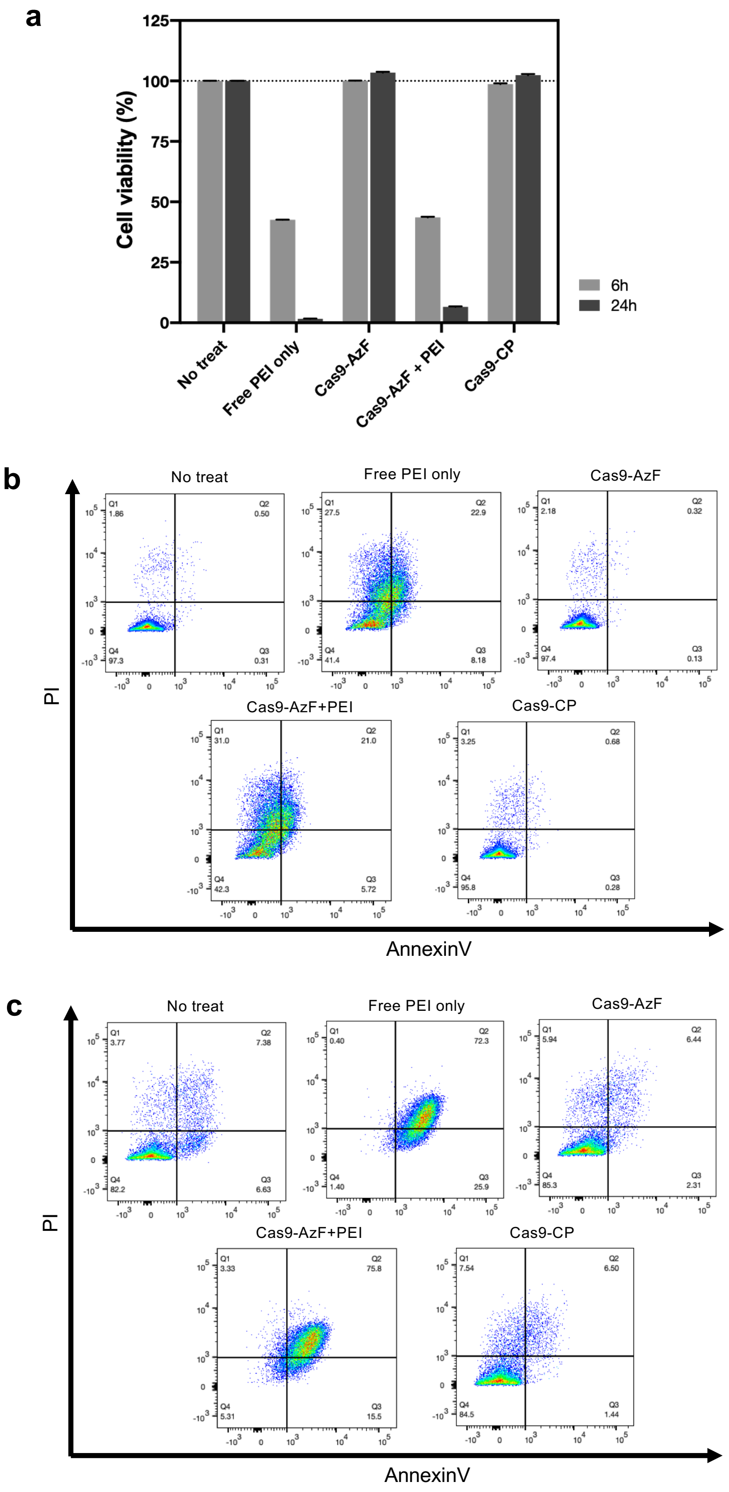

Figure S8. Examination of cytotoxicity of Cas9-CP conjugates treated to HCC1937 cells, compared with free polyethylenimine (PEI) complexed with Cas9-AzF RNP (free PEI concentration: 36 µg/ml). (a) Quantitative data of cell viabilities. (b,c) Flow cytometry plots of cells treated for (b) 6 h, and (c) 24 h.

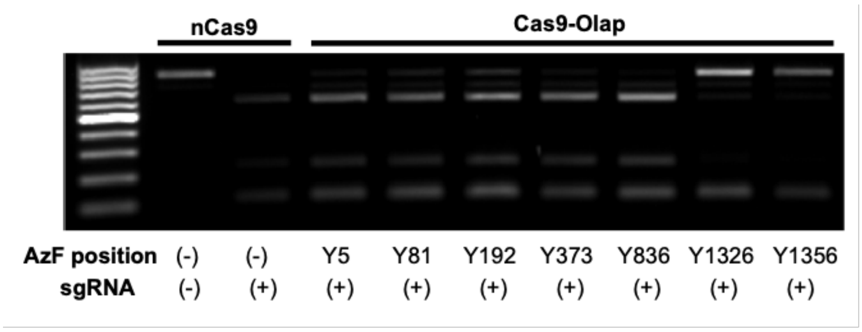

Figure S9. Cleavage activities of Cas9-Olap conjugates for different Cas9-AzF variants. Cas9-Olap conjugates were prepared by reacting the azido group of Cas9-AzF with DBCO-functionalized olaparib.

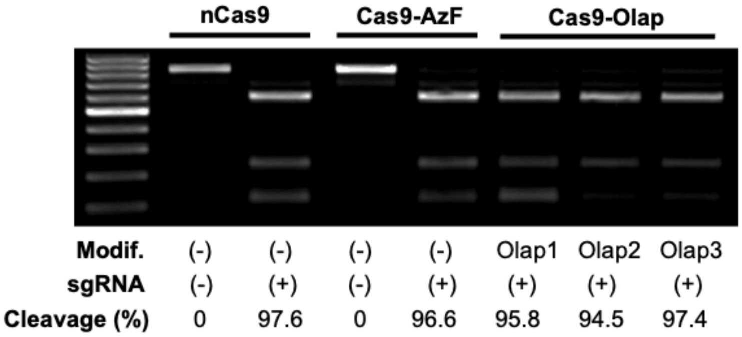

Figure S10. Cleavage activities of Cas9-Olap conjugates prepared via different functional groups. Cas9-Olap conjugates were prepared by reacting Cas9-AzF with olaparib via azido groups of AzF (Olap1), sulfhydryl groups of cysteine (Olap2), or both azido and sulfhydryl groups (Olap3).

Treatment	Cell line	Indel freq. (%)
Cas9-AzF	HEK293	20.8
	MDA-MB-231	25.1
Cas9-Olap	HEK293	23.3
	MDA-MB-231	23.2

Figure S11. RAD52 gene editing efficiencies by Cas9-Olap conjugates in control cell lines. Cas9-Olap RNPs was prepared with the Y373 variant conjugated with olaparib via azido and sulfhydryl groups (Cas9-Olap3), and complexed with RAD52 sgRNA (1:1). RNPs complexed with CMAX were treated to HEK293T and MDA-MB-231 cells for 48 h at 500 nM, and analyzed by targeted deep sequencing.

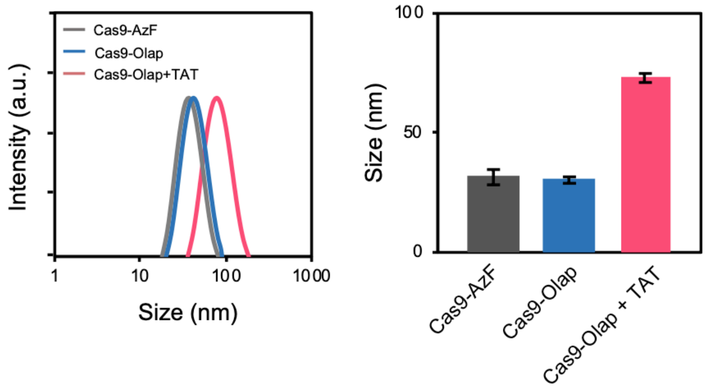

Figure S12. DLS measurements of Cas9-Olap conjugate RNPs complexed with TAT peptide. Histogram (left) and average sizes (right). Molar ratio of Cas9:TAT = 1:10.

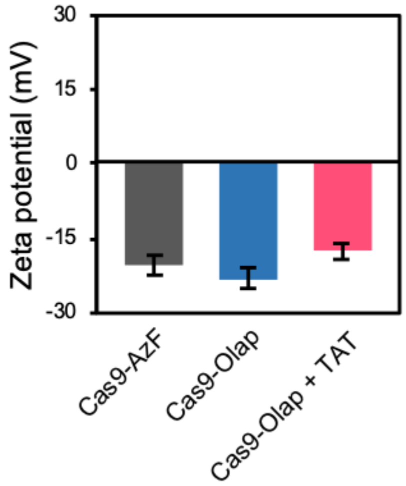

Figure S13. Zeta potential measurements of Cas9-Olap conjugate RNPs complexed with TAT peptide (molar ratio of Cas9:TAT = 1:10).

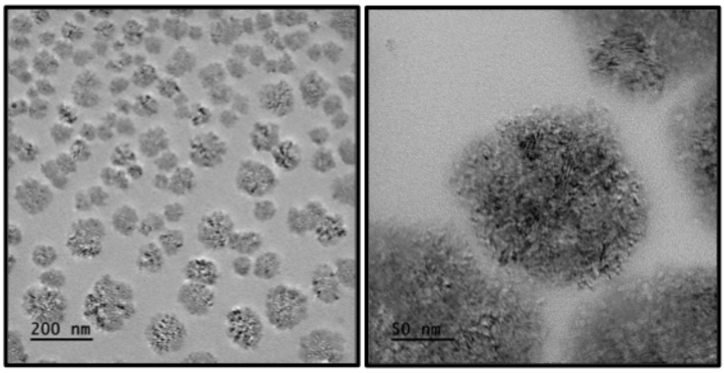

Figure S14. TEM of Cas9-Olap conjugate RNPs complexed with TAT peptide (molar ratio of Cas9:TAT = 1:10).

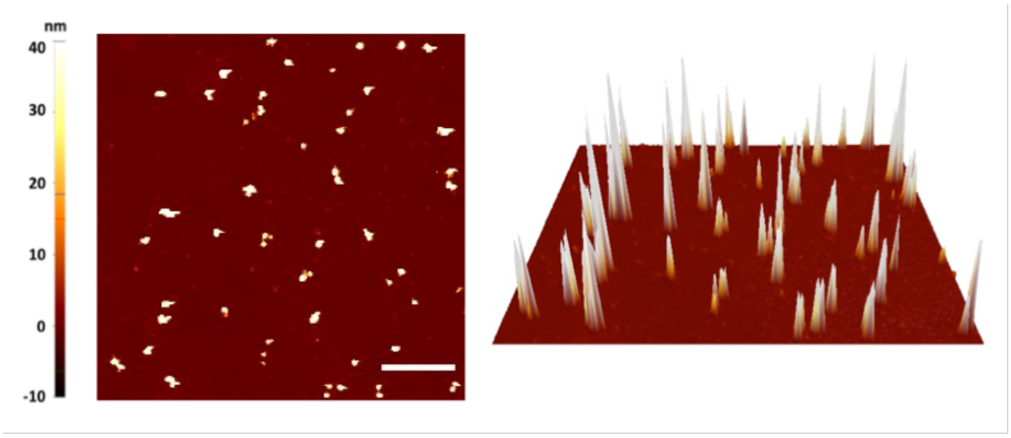

Figure S15. AFM of Cas9-Olap conjugate RNPs complexed with TAT peptide (molar ratio of Cas9:TAT = 1:10).

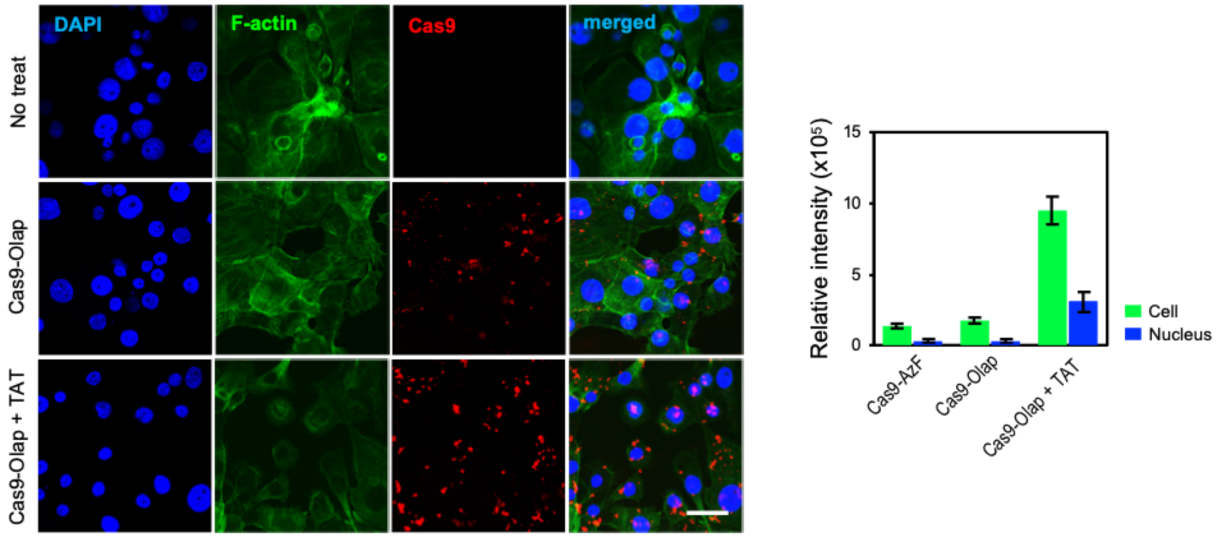

Figure S16. Cellular uptake of Cas9-Olap conjugates RNPs complexed with TAT peptide. HCC1937 cells were treated with the RNP/TAT complexes (molar ratio of Cas9:TAT = 1:10) for 4 h.

a

Treatment	Cell line	Indel freq. (%)
nCas9	HCC1937	15.7
Cas9-AzF	HCC1937	13.9
	MDA-MB-231	17.6
Cas9-Olap	HCC1937	15.9
	MDA-MB-231	18.1

b

	RAD52 gene	Indel type	Indel (%)
WT	GGGCCCAGAATACATAAGTA GCC GCATGGCTGGCGGAGGCCAGAAGGT		
#1	GGGCCCAGAATACATAAGTA - - - GCATGGCTGGCGGAGGCCAGAAGGT	-3	3.75
#2	GGGCCCAGAATACATAAGTAGCCCGCATGGCTGGCGGAGGCCAGAAGGT	+1	2.69
#3	GGGCCCAGAATACATAAGTAG - CGCATGGCTGGCGGAGGCCAGAAGGT	-1	1.62
#4	GGGCCCAGAATACATAAGT - - - CGCATGGCTGGCGGAGGCCAGAAGGT	-3	0.95

Figure S17. Gene editing efficiencies by Cas9-Olap conjugate RNPs complexed with TAT peptide. Complexes (molar ratio of Cas9:TAT = 1:10) were treated to cells for 4 h, and analyzed by targeted deep sequencing. (a) Indel frequencies of HCC1937 and MDA-MB-231 cells after treatment. (b) Representative sequences of HCC1937 cells treated with Cas9-Olap RNPs.

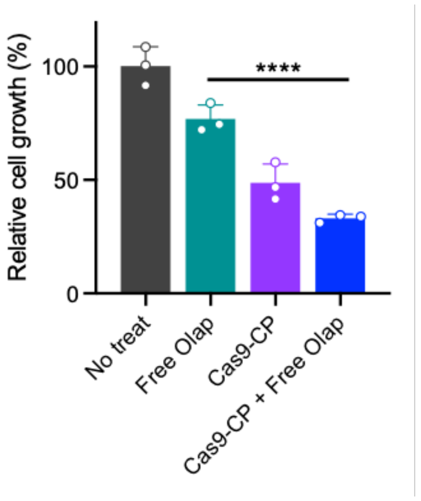

Figure S18. Anti-proliferation effect upon treatment of free olaparib, Cas9-CP RNPs, and their mixture. Complexes and controls were treated to HCC1937 cells for 72 h. Cas9-CP conjugates were prepared by reacting the sulfhydryl groups of Cas9 with CP, complexed with sgRNA (1:1), and treated at 500 nM.

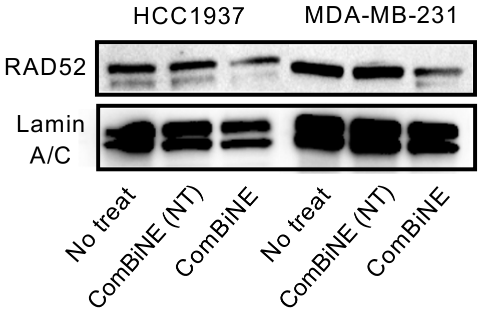

Figure S19. Western blot analysis of RAD52 expression in HCC1937 and MDA-MB-231 cells treated with ComBiNE. Cas9-Olap1-CP2 conjugates were complexed with RAD52 or non-target (NT) sgRNA (1:1), and treated to cells for 48 h at 500 nM.

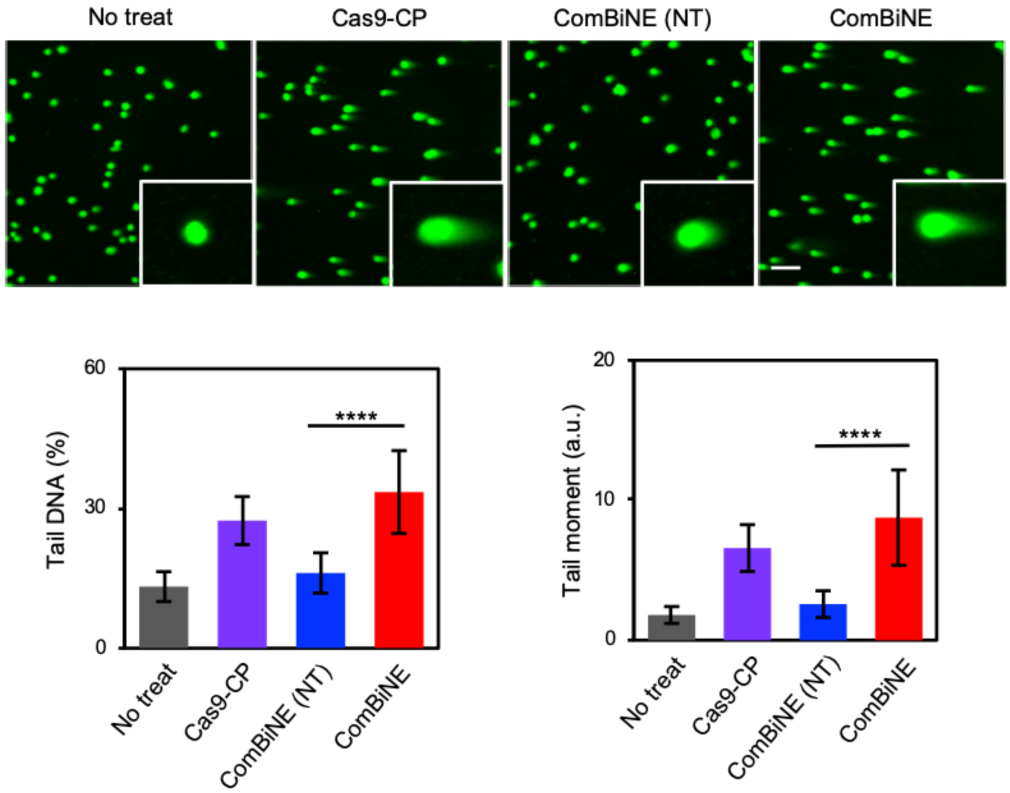

Figure S20. Comet assay of MDA-MB-231 cells treated with ComBiNE and control formulations. Treatment at 500 nM Cas9 for 48 h. $^{***}P < 0.001$.

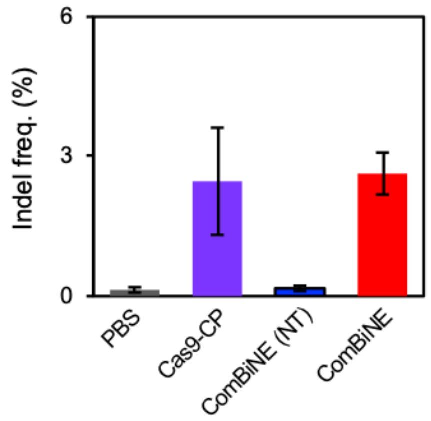

Figure S21. Indel frequencies of mice tumors at 24 days post-treatment. Mice with HCC1937 tumors were treated with ComBiNE and control formulations, and tissues were harvested at day 24, followed by targeted deep sequencing (*n* = 3).

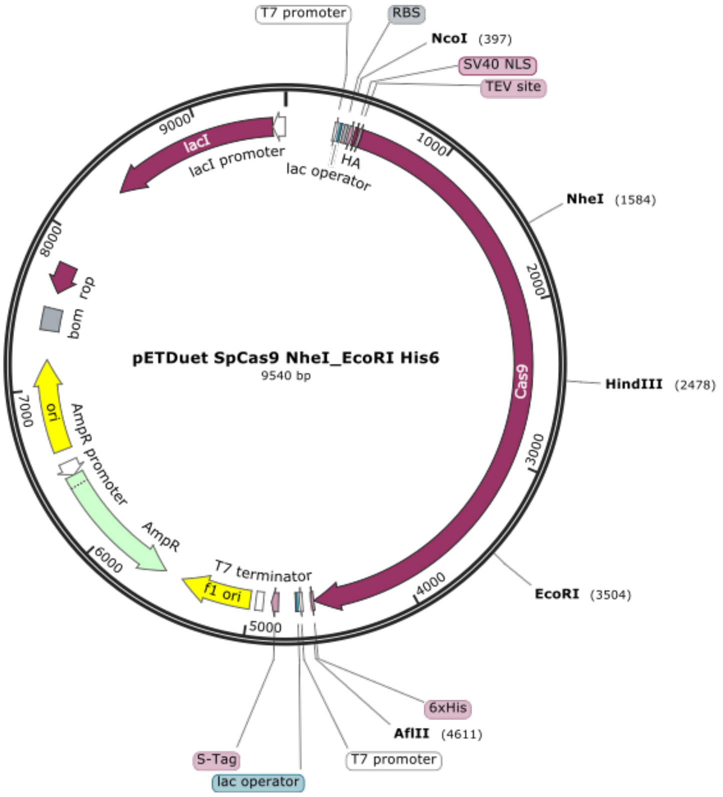

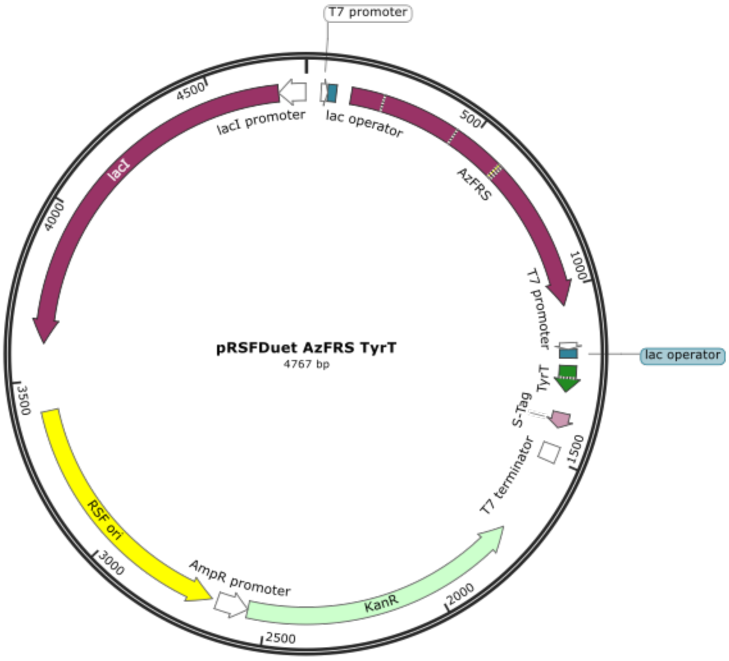

Figure S22. Plasmid maps. pETDuet SpCas9 wt His6 (top) and pRSFDuet AzFRS TyrT (bottom).
